# Implications of systolic pulmonary artery pressure trajectories in systemic lupus erythematosus-associated pulmonary hypertension: a CSTAR-PAH cohort study

**DOI:** 10.3389/fimmu.2026.1819805

**Published:** 2026-06-09

**Authors:** Qifang Guo, Dunwu Wu, Xiuling Zhang, Le Yu, Jingjing Shang, Xueqin Feng, Rongwei Zhang, Shaoyuan Mao, Wei Zhou, Xinwang Duan

**Affiliations:** Department of Rheumatology and Immunology, the Second Affiliated Hospital, Jiangxi Medical College, Nanchang University, Nanchang, Jiangxi, China

**Keywords:** group-based trajectory modelling, mortality, prognosis, pulmonary hypertension, systemic lupus erythematosus

## Abstract

**Objective:**

To identify the evolution trajectory of systolic pulmonary artery pressure (sPAP) in patients with systemic lupus erythematosus-related pulmonary arterial hypertension (SLE-PAH) and to evaluate its prognostic significance.

**Method:**

In this study, 81 SLE-PAH patients from the Chinese SLE Treatment and Research Group-Pulmonary Arterial Hypertension (CSTAR-PAH) cohort of the Second Affiliated Hospital of Nanchang University were included for a retrospective cohort study. A group-based trajectory model was used to identify the trajectories of sPAP over time. Baseline factors related to trajectory assignment were analyzed through multiple logistic regression. Univariate and multivariate Cox regression and Kaplan–Meier analysis were used to evaluate the prognostic value of trajectory groups for all-cause mortality, and to analyze the related influencing factors of all-cause mortality.

**Results:**

Four distinct sPAP trajectories were identified: trajectory 1 (n = 17, 20.9%; high initial sPAP with rapid decrease), trajectory 2 (n = 44, 54.3%; moderate initial sPAP with slow decrease), trajectory 3 (n = 13, 16.0%; low initial sPAP with slow decrease), and trajectory 4 (n = 7, 8.6%; high initial sPAP with rapid increase). Longer 6-minute walking distance (6MWD) was independently associated with trajectories 2 (OR = 3.077, p < 0.001) and 3 (OR = 11.106, p < 0.001), whereas shorter 6MWD (OR = 0.220, p < 0.001), elevated B-type natriuretic peptide (OR = 2.159, p < 0.001), higher IL-1β (OR = 1.412, p < 0.001), higher TNF-α (OR = 1.751, p < 0.001), and higher mean pulmonary artery pressure (OR = 1.067, p < 0.001) independently predicted trajectory 4 membership. During follow-up, trajectory 4 remained a strong independent predictor of mortality (HR = 8.843, p = 0.037) after multivariable adjustment, together with higher systemic lupus erythematosus disease activity index (HR = 1.502, p = 0.032), elevated IL-6 (HR = 1.031, p=0.029), higher systemic inflammation response index (HR = 1.828, p = 0.019), and shorter 6MWD (HR = 0.795, p = 0.028). Patients in trajectory 4 exhibited significantly worse survival (log-rank p < 0.0001).

**Conclusion:**

sPAP trajectories capture clinically meaningful heterogeneity in SLE-PAH, with the rapid−progression trajectory identifying a high−risk phenotype characterized by pronounced inflammation, right ventricular strain, and functional compromise. Trajectory−based phenotyping enhances risk stratification and may guide personalized management in SLE-PAH.

## Introduction

Systemic lupus erythematosus (SLE) is a complex chronic autoimmune disease characterized by multiple-organ involvement and persistent immune dysregulation. Among the cardiovascular and pulmonary manifestations of this disease, pulmonary arterial hypertension (PAH) is one of the most serious and life-threatening complications. SLE-associated PAH (SLE-PAH) significantly increases the incidence and mortality rate and remains a major determinant of long-term prognosis. Epidemiological studies have confirmed that PAH is not uncommon in SLE patients. A single-center prospective cohort study reported that approximately 7.96% of SLE patients developed PAH during the follow-up period (1). The prospective cohort data from the Chinese SLE Treatment and Research Group (CSTAR) further showed that the prevalence of SLE-PAH in Chinese patients was approximately 3.8% (2). Although advances in immunosuppressive and targeted PAH therapies have improved outcomes, survival remains significantly worse in SLE-PAH compared with SLE patients without pulmonary vascular involvement. Despite the 5-year survival rate of general SLE populations ranging from 92% to 94%, the reported 5-year overall survival of SLE-PAH patients varies between 68% and 84% (3, 4). These data highlight the urgent need for improved risk stratification strategies in this high-risk subgroup.

Mean pulmonary arterial pressure (mPAP), measured by right heart catheterization (RHC), remains the cornerstone hemodynamic parameter for the diagnosis and severity assessment of PAH. The updated hemodynamic definition proposed at the 6th World Symposium on Pulmonary Hypertension defines pulmonary hypertension as mPAP >20 mmHg at rest (5). Although mean pulmonary artery pressure (mPAP) not only has diagnostic value but also reflects the degree of pulmonary vascular remodeling and the right ventricular afterload (a key prognostic indicator), the premise of relying solely on a single baseline measurement is relative hemodynamic stability, which may not accurately reflect the dynamic evolution of systemic lupus erythematosus pulmonary hypertension (SLE-PAH). Emerging evidence supports the superior prognostic value of serial biomarker trajectories, including hemodynamic indices, over static assessments (6, 7). In idiopathic PAH, longitudinal mPAP trends correlate strongly with long-term outcomes, underscoring the need for dynamic rather than point-in-time hemodynamic evaluation in pulmonary vascular disease (8, 9).

Nevertheless, the practical implementation of longitudinal hemodynamic monitoring remains challenging. Although RHC can provide clear assessment results, its invasiveness, operational risks, cost, and the feasibility of repeated examinations are limited, which restricts its routine application in close longitudinal monitoring, especially for patients with systemic autoimmune diseases who require long-term follow-up and continuous monitoring. In real-world clinical practice, serial RHC measurements are seldom performed at short intervals, thereby limiting the ability to fully characterize hemodynamic trajectories (7). Transthoracic echocardiography, in contrast, offers a non-invasive, repeatable, and widely accessible modality for estimating pulmonary artery pressure. Current guidelines from the European Society of Cardiology and European Respiratory Society recommend echocardiography as the primary screening and follow-up tool in suspected pulmonary hypertension (10). Validation studies have demonstrated significant correlations between echocardiographically estimated pulmonary artery pressures and invasively measured hemodynamic parameters (11). Importantly, because echocardiography can be safely repeated over time, it enables serial estimation of mPAP during long-term follow-up, thereby providing a pragmatic foundation for trajectory-based hemodynamic phenotyping in routine clinical practice. Nevertheless, longitudinal hemodynamic data specific to SLE-PAH remain scarce. This gap is particularly relevant given the unique pathophysiology of SLE-PAH, which integrates immune-mediated endothelial injury with classical pulmonary vasculopathy (12). Fluctuations in autoimmune activity and variable responsiveness to immunosuppressive therapy may substantially influence pulmonary pressure evolution. Therefore, characterizing the trajectory of mPAP over time may uncover distinct clinical phenotypes and inform personalized immunomodulatory and vasodilator treatment strategies.

Understanding the longitudinal patterns and prognostic significance of mPAP trajectories in SLE-PAH may improve risk stratification beyond baseline hemodynamic assessment. Dynamic profiling of pulmonary pressure could facilitate early identification of patients at increased risk of mortality despite initial therapy and support timely treatment intensification. In this study, we utilized serial echocardiography-derived measurements to identify distinct longitudinal mPAP trajectory patterns in patients with systemic lupus erythematosus–associated pulmonary arterial hypertension (SLE-PAH). We further compared baseline clinical characteristics, inflammatory profiles, and potential determinants across trajectory groups and evaluated the independent association between trajectory membership and all-cause mortality, while exploring additional independent predictors of death.

## Methods

### Patients

Based on data obtained from the Chinese SLE Treatment and Research Group-Pulmonary Arterial Hypertension (CSTAR-PAH) cohort between March 2014 and January 2026, a retrospective study was conducted comprising patients with systemic lupus erythematosus–associated pulmonary arterial hypertension (SLE-PAH) who were managed at participating CSTAR centers during the study period. SLE was diagnosed by experienced rheumatologists according to either the 2012 Systemic Lupus International Collaborating Clinics (SLICC) classification criteria or the 2019 European League Against Rheumatism/American College of Rheumatology (EULAR/ACR) classification criteria (13). The diagnosis of PAH was confirmed by right heart catheterization (RHC), defined as a mean pulmonary arterial pressure (mPAP) >20 mmHg at rest, pulmonary arterial wedge pressure (PAWP) ≤15 mmHg, and pulmonary vascular resistance (PVR) >2 Wood units (14, 15).

Patients were excluded if pulmonary hypertension was not attributable to SLE. Specifically, individuals with significant parenchymal lung disease indicated by a total lung capacity (TLC) <60% on pulmonary function testing, or with evidence of chronic thromboembolic disease demonstrated by ventilation–perfusion scintigraphy or computed tomographic pulmonary angiography (CTPA), were excluded. Patients diagnosed with other forms of pulmonary hypertension were also not eligible. In addition, those with overlapping connective tissue diseases were excluded from the analysis. To ensure the reliability of longitudinal trajectory modeling, patients with incomplete clinical records, missing key variables, or fewer than three echocardiography-derived mPAP assessments within the first 2 years of follow-up were excluded. The study was conducted in accordance with local regulations and approved by the Medical Ethics Committee of the Second Affiliated Hospital of Nanchang University.

### Data collection

The data used in this study were obtained from the CSTAR-PAH cohort and patients’ medical records. The time of recruitment was defined as the time of SLE-associated pulmonary arterial hypertension (PAH) diagnosis confirmed by right heart catheterization (RHC) at our center (baseline). The primary endpoint was death from any cause. The occurrence of death and the duration from PAH diagnosis to the event were recorded. The follow-up period for individual patients was from the date of PAH diagnosis (baseline) through death, loss to follow-up, or January 2026.

Essential characteristic, risk stratification parameters, clinical feature, laboratory parameters, inflammatory variables, serology and autoantibodies, lymphocyte subsets, cytokines, pulmonary function tests, and treatment were collected. The specific variables are shown in **Table 1**. This study recorded serial assessments of systolic pulmonary artery pressure (sPAP) obtained through echocardiography. The sPAP was derived from the sum of two components: the right ventricular to right atrial systolic pressure gradient, calculated via the simplified Bernoulli equation based on the peak tricuspid regurgitation velocity (TRV), and the right atrial pressure, which was estimated from the diameter and respiratory collapsibility of the inferior vena cava (14). Screening echocardiography was performed every 3 months during the first year of follow-up and then as appropriate during the second year. Missing variables were handled with the use of multiple imputation, as shown in **Supplementary Table 1**. The data underlying this study are not publicly available because they are derived from an ongoing research project. Requests for access to the data may be directed to the corresponding author.

**Table 1:** Baseline characteristics at study inclusion of the cohort with systemic lupus erythematosus-associated pulmonary arterial hypertension (SLE-PAH).

Characteristics	Entire cohort (N = 81)^1^
Essential characteristic	
Age (y)	45.00 (37.00, 54.00)
Gender	
Male	6 (7.4%)
Female	75 (92.6%)
Disease duration (m)	42.00 (24.00, 60.00)
BMI	19.80 (18.20, 21.64)
Trajectory 4 group	
1	17 (21.0%)
2	44 (54.3%)
3	13 (16.0%)
4	7 (8.6%)
Death	8 (9.9%)
Baseline mPAP (mmHg)	54.00 (45.00, 67.00)
Risk stratification parameters	
WHO functional class	
I	19 (23.5%)
II	29 (35.8%)
III	19 (23.5%)
IV	14 (17.3%)
6MWD (m)	42.50 (33.00, 45.90)
SLEDAI	6.00 (4.00, 9.00)
Clinical feature	
Acute subacute cutaneous lupus	20 (24.7%)
Chronic cutaneous lupus	8 (9.9%)
Oral ulcers	3 (3.7%)
Non-scarring alopecia	14 (17.3%)
Arthritis	27 (33.3%)
hemolyticHemolytic anemia	8 (9.9%)
Leukopenia	29 (35.8%)
Thrombocytopenia	33 (40.7%)
TTP	1 (1.2%)
Serositis	26 (32.1%)
Lupus nephritis	23 (28.4%)
Interstitial lung disease	11 (13.6%)
Gastrointestinal	2 (2.5%)
Neuropathy	5 (6.2%)
Laboratory parameters	
WBC (×10^9^/L)	5.36 (3.54, 7.52)
Hb (g/L)	106.00 (93.00, 124.00)
PLT (×10^9^/L)	162.00 (111.00, 225.00)
Neutrophil count (×10^9^/L)	3.76 (2.24, 6.32)
Lymphocyte count (×10^9^/L)	1.19 (0.74, 1.76)
Monocyte count (×10^9^/L)	0.32 (0.10, 0.70)
Albumin (g/L)	33.97 (30.88, 40.03)
Total bilirubin (μmol/L)	8.85 (7.15, 13.68)
Direct bilirubin (μmol/L)	2.39 (1.59, 4.16)
AST (U/L)	24.29 (19.40, 32.84)
ALT (U/L)	14.17 (10.80, 24.88)
LDH (U/L)	3.79 (2.12, 219.00)
Uric acid (μmol/L)	7.53 (4.58, 336.67)
Creatinine (μmol/L)	63.70 (51.10, 72.94)
eGFR (mL/min/1.73m²)	96.23 (79.93, 122.81)
BNP (pg/mL)	182.20 (72.60, 511.00)
Inflammatory variables	
ESR (mm/h)	46.00 (24.00, 86.00)
CRP (mg/L)	5.72 (2.61, 24.50)
SII	575.88 (189.13, 1,067.25)
SIRI	0.96 (0.25, 2.15)
NLR	3.00 (1.91, 5.49)
Serology and autoantibodies	
IgG (g/L)	19.85 (15.60, 27.20)
IgA (g/L)	3.54 (2.48, 4.37)
IgM (g/L)	1.27 (0.81, 1.78)
C3 (g/L)	0.60 (0.40, 0.78)
C4 (g/L)	0.13 (0.07, 0.18)
ANA titer	
0	3 (3.7%)
100	3 (3.7%)
160	6 (7.4%)
320	9 (11.1%)
640	1 (1.2%)
800	1 (1.2%)
1,000	8 (9.9%)
3,200	49 (60.5%)
6,400	1 (1.2%)
Anti dsDNA (IU/mL)	52.00 (7.20, 196.00)
ANA C1q	2.42 (0.77, 8.87)
Anti-Sm	0.21 (0.10, 0.76)
Anti-nRNP	3.41 (0.30, 5.76)
Anti-SSA	3.15 (1.11, 4.08)
Anti-Ro52	2.63 (0.68, 4.45)
Anti-SSB	0.16 (0.04, 0.75)
Anti-Scl70	0.12 (0.05, 0.41)
Anti-PM Scl	0.11 (0.05, 0.34)
Anti-Jo1	0.11 (0.05, 0.39)
Anti-ANUA	0.42 (0.16, 1.91)
Anti-CENPB	0.04 (0.01, 0.29)
Anti-histone	0.32 (0.13, 1.41)
Anti-RIB P	0.20 (0.04, 0.97)
Anti-AMA M2	0.25 (0.06, 0.59)
Lupus anticoagulant ratio	1.12 (1.01, 1.29)
aCL IgA	2.50 (2.50, 2.50)
aCL IgG	4.10 (2.60, 6.50)
aCL IgM	2.00 (2.00, 2.10)
Anti-β2 glycoprotein IgA	2.00 (2.00, 3.40)
Anti-β2 glycoprotein IgG	3.00 (2.00, 5.10)
Anti-β2 glycoprotein IgM	2.00 (2.00, 2.40)
Lymphocyte subsets	
T-cell percent	74.55 (66.34, 79.99)
T helper percent	30.50 (22.75, 36.00)
T suppressor percent	37.76 (28.49, 46.00)
NK cell percent	8.94 (5.40, 15.26)
B cell percent	10.87 (4.34, 16.75)
DPT cell percent	0.36 (0.17, 0.63)
DNT cell percent	3.95 (2.76, 5.54)
NK T-cell percent	0.77 (0.66, 1.17)
T-cell count	719.00 (475.00, 993.00)
T helper count	260.19 (186.00, 362.74)
T suppressor count	366.99 (215.52, 560.00)
NK cell count	88.00 (54.00, 140.00)
B cell count	75.46 (44.00, 115.01)
DPT cell count	2.00 (2.00, 4.77)
DNT cell count	28.00 (20.00, 50.00)
NKT cell count	20.97 (19.51, 24.00)
TBNK percent	98.79 (98.68, 99.03)
TBNK count	819.90 (765.69, 855.31)
THTS ratio	0.82 (0.61, 1.23)
Cytokines (pg/mL)	
IL-5	2.10 (1.48, 2.32)
IFN-α	1.98 (1.63, 2.68)
IL-2	1.78 (1.54, 2.11)
IL-6	4.09 (2.36, 10.98)
IL-1β	6.04 (2.80, 9.69)
IL-10	2.26 (1.74, 2.82)
IFN-γ	5.80 (2.21, 13.74)
IL-8	4.06 (2.10, 8.65)
IL-17	3.54 (2.11, 4.91)
IL-4	1.26 (0.95, 1.68)
IL-12p70	1.66 (1.34, 2.02)
TNF-α	2.11 (1.90, 2.93)
Pulmonary function tests	
FVC percent	67.50 (65.60, 71.20)
FEV1	67.55 (65.00, 69.10)
TLC	78.15 (78.15, 78.15)
DLCO	49.80 (49.80, 49.80)
Treatment	
Glucocorticoids	78(96.3%)
High dose	35(43.2%)
Medium dose	29(35.8%)
Low dose	14(17.28%)
Immunosuppressants	
Hydroxychloroquine sulfate	68(84.0%)
Cyclophosphamide	24(29.6%)
Mycophenolate mofetil	29(25.8%)
Ciclosporin A	5(6.2%)
Tacrolimus	5(6.2%)
Azathioprine	4(4.9%)
Methotrexate	2(2.47%)
Iguratimod	1(1.23%)
PAH treatment regimen	
Endothelin receptor antagonist	23(28.0%)
Phosphodiesterase 5 inhibitor	22(27.2%)
Prostaglandin analogs	2(2.5%)

^1^ Mean (SD); median (IQR); n (%). BMI, body mass index; mPAP, mean pulmonary artery pressure; 6MWD, 6-minute walking distance; SLEDAI, Systemic Lupus Erythematosus Disease Activity Index; TTP, thrombotic thrombocytopenic purpura; ILD, interstitial lung disease; WBC, white blood cell count; Hb, hemoglobin; PLT, platelet; AST, aspartate aminotransferase; ALT, alanine aminotransferase; LDH, lactate dehydrogenase; eGFR, estimated glomerular filtration rate; BNP, brain natriuretic peptide; ESR, erythrocyte sedimentation rate; CRP, C-reactive protein; SII, systemic immune-inflammation index; SIRI, systemic inflammation response index; NLR, neutrophil-to-lymphocyte ratio; IgG, immunoglobulin G; IgA, immunoglobulin A; IgM, immunoglobulin M; C3, complement 3; C4, complement 4; ANA, antinuclear antibody; anti-dsDNA, anti-double-stranded DNA antibody; anti-Sm, anti-Smith antibody; anti-nRNP, anti-ribonucleoprotein antibody; anti-SSA, anti-Sjögren’s syndrome antigen A antibody; anti-Ro52, anti-Ro52 antibody; anti-SSB, anti-Sjögren’s syndrome antigen B antibody; anti-Scl70, anti-topoisomerase I antibody; anti-PM-Scl, anti-polymyositis-scleroderma antibody; anti-Jo1, anti-histidyl-tRNA synthetase antibody; anti-ANUA, anti-nucleosome antibody; anti-CENPB, anti-centromere protein B antibody; anti-histone, anti-histone antibody; anti-RIB P, anti-ribosomal P protein antibody; anti-AMA-M2, anti-mitochondrial antibody M2; LAC, lupus anticoagulant; aCL, anticardiolipin antibody; anti-β2GPI, anti-β2 glycoprotein I antibody; NK cell, natural killer cell; DPT cell, double-positive T cell; DNT cell, double-negative T cell; NKT cell, natural killer T cell; TBNK, T cells, B cells, and NK cells; THTS, T helper/T suppressor ratio; IL-5, interleukin-5; IFN-α, interferon-alpha; IL-2, interleukin-2; IL-6, interleukin-6; IL-1β, interleukin-1β; IL-10, interleukin-10; IFN-γ, interferon-gamma; IL-8, interleukin-8; IL-17, interleukin-17; IL-4, interleukin-4; IL-12p70, interleukin-12p70; TNF-α, tumor necrosis factor-alpha; FVC, forced vital capacity; FEV1, forced expiratory volume in 1 second; TLC, total lung capacity; DLCO, diffusing capacity of the lung for carbon monoxide; MMF, mycophenolate mofetil; ERA, endothelin receptor antagonist; PDE5i, phosphodiesterase type 5 inhibitor.

### Trajectory analysis of longitudinal sPAP changes

Longitudinal patterns of sPAP, estimated by transthoracic echocardiography, were examined using group-based trajectory modeling (GBTM). This semi-parametric finite mixture approach identifies latent subgroups of individuals who share similar developmental trends over time and accommodates heterogeneity in longitudinal change within observational cohorts (16). A series of candidate models were specified with two to six trajectory groups. Within each model, trajectory shapes were parameterized using polynomial functions up to the third order. Model adequacy was evaluated according to recommended criteria: (1) average posterior probability of group membership ≥0.70 for each trajectory; (2) odds of correct classification ≥5.0; (3) relative entropy ≥0.50; and (4) a minimum group size of >3% of the total cohort to ensure stability and clinical relevance. Among models satisfying these criteria, the final model was selected based on the Bayesian Information Criterion (BIC) value in conjunction with clinical interpretability of the number and shape of the trajectories.

### Statistical analysis

Continuous variables were assessed for normality prior to analysis. Normally distributed data are presented as mean ± standard deviation (SD), whereas non-normally distributed variables are expressed as median with interquartile range (IQR). Categorical variables are summarized as counts and percentages. Between-group comparisons were conducted according to data distribution. Continuous variables were analyzed using the independent-samples t test or the Mann–Whitney U test, as appropriate. Categorical variables were compared using the χ² test or Fisher’s exact test when expected cell counts were small. Time-to-event outcomes were analyzed using Kaplan–Meier survival curves. In analyses where death was considered a competing event, cumulative incidence functions were applied to account for competing risk effects.

Associations between baseline clinical characteristics during the first 2 years after disease onset and trajectory group membership were examined using multinomial logistic regression, with clinical variables entered as independent predictors and trajectory assignment as the dependent outcome. The relationship between longitudinal sPAP trajectory patterns and all-cause mortality was evaluated using Cox proportional hazards regression models. Results are reported as hazard ratios (HRs) with corresponding 95% confidence intervals (CIs). To enhance the stability of the Cox proportional hazards model for all-cause mortality and minimize potential overfitting, the least absolute shrinkage and selection operator (LASSO) approach was applied to identify the most informative predictors from the prespecified candidate variables (17). This penalized regression technique improves model robustness by shrinking regression coefficients and selecting variables with the strongest prognostic contribution. All statistical analyses were performed using R software (version 3.4.3; http://www.R-project.org/). A two-sided P value < 0.05 was considered statistically significant.

## Results

### Patient characteristics

A total of 81 patients with SLE-PAH were included in the analysis. The baseline demographics and clinical characteristics of this cohort are summarized in **Table 1**. The study population was predominantly female, comprising 75 patients (92.6%), with a median age of 45.0 years (IQR: 37.0–54.0). The median disease duration of SLE-PAH was 42.0 months (IQR: 24.0–60.0), and the median body mass index (BMI) was 19.80 kg/m² (IQR: 18.20–21.64). Regarding functional status, 48 patients (59.3%) presented with WHO functional class (FC) I–II symptoms at baseline, including 19 (23.5%) in FC I and 29 (35.8%) in FC II. The median 6-minute walk distance (6MWD) was 42.5 m (IQR: 33.0–45.9). Disease activity, as assessed by the SLEDAI, yielded a median score of 6.0 (IQR: 4.0–9.0). Hemodynamically, the median baseline mean pulmonary arterial pressure (mPAP) was 54.0 mmHg (IQR: 45.0–67.0). Laboratory evaluations revealed a median B-type natriuretic peptide (BNP) level of 182.20 ng/L (IQR: 72.60–511.00). Inflammatory markers were elevated, with a median erythrocyte sedimentation rate (ESR) of 46.0 mm/h (IQR: 24.0–86.0) and a median C-reactive protein (CRP) level of 5.72 mg/L (IQR: 2.61–24.50). Immunological profiling showed median complement C3 and C4 levels of 0.60 g/L (IQR: 0.40–0.78) and 0.13 g/L (IQR: 0.07–0.18), respectively, and a median anti-dsDNA antibody titer of 52.00 IU/mL (IQR: 7.20–196.00). In terms of therapeutic interventions, the vast majority of patients (78, 96.3%) received glucocorticoid therapy. Based on the administered dosage, patients were categorized into three groups: high-dose (>1.0 mg/kg/day) in 35 patients (43.2%), medium-dose (0.5–1.0 mg/kg/day) in 29 patients (35.8%), and low-dose (<0.5 mg/kg/day) in 14 patients (17.3%). Immunosuppressive agents were employed in a significant proportion of the cohort. Hydroxychloroquine was the most frequently used immunosuppressant, prescribed in 68 patients (84.0%), followed by cyclophosphamide in 24 patients (29.6%) and mycophenolate mofetil in 29 patients (25.8%). Regarding targeted PAH therapy, 23 patients (28.0%) were treated with endothelin receptor antagonists (ERAs), 22 patients (27.2%) received phosphodiesterase type 5 inhibitors (PDE-5is), and 2 patients (2.5%) were on prostaglandin analogs.

### sPAP trajectories

Group-based trajectory modeling was employed to identify distinct trajectories of sPAP over time. When fixing the polynomial order at three, we exploratorily fitted trajectory models with one to six groups; the model fit indices are presented in **Table 2**. Both BIC and AIC decreased progressively with an increasing number of groups. However, when the number of groups exceeded four, the magnitude of decline in BIC and AIC began to diminish markedly, and the BIC increased when a six-group model was fitted. Considering clinical interpretability and model parsimony, the four-group model was ultimately determined as the most appropriate for this cohort.

**Table 2:** Fit statistics and class membership for the trajectory models.

Model	APP						AIC	BIC	CAIC	SSBIC	HQIC
	G1	G2	G3	G4	G5	G6					
traj_1	1	NA	NA	NA	NA	NA	4,986.510307	5,008.212103	5,013.212103	4,992.33944	4,994.979661
traj_2	0.982516196	0.988130835	NA	NA	NA	NA	4,583.59771	4,622.660944	4,631.660944	4,594.090149	4,598.842548
traj_3	0.950600509	0.983944241	0.999999951	NA	NA	NA	4,480.550614	4,554.336722	4,571.336722	4,500.369666	4,509.346419
traj_4	0.969192607	0.987024563	0.997931351	0.999999955	NA	NA	4,359.404231	4,459.232495	4,482.232495	4,386.218243	4,398.363261
traj_5	0.994730937	0.970002463	0.957703322	0.999101135	0.999999801	NA	4,316.348106	4,442.218526	4,471.218526	4,350.157078	4,365.470362
traj_6	0.997075043	0.942054052	0.925931084	0.999770065	0.999999909	0.983883281	4,297.378267	4,449.290842	4,484.290842	4,338.182198	4,356.663748

traj_1, trajectory model 1;traj_2, trajectory model 2;traj_3, trajectory model 3;traj_4, trajectory model 4;traj_5, trajectory model 5;traj_6, trajectory model 6;APP, posterior probability of assignments; AIC, Akaike Information Criterion; BIC, Bayesian Information Criterion; CAIC, Consistent Akaike Information Criterion; SSBIC, Sample-Size Adjusted Bayesian Information Criterion; HQIC, Hannan Quinn Information Criterion.

**Figure 1:**
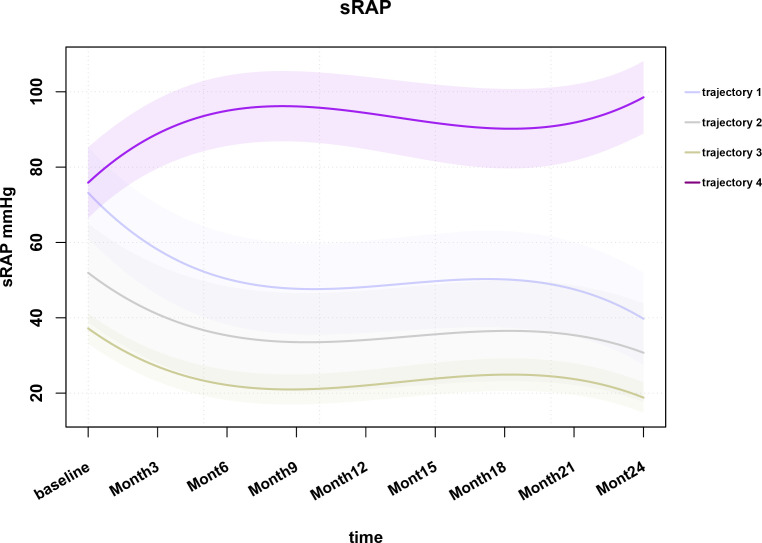
sPAP trajectories over time in SLE-PAH patients. Data are presented as median sPAP values (mmHg) for each trajectory group during 24 months of follow-up. Trajectory 1 (n=17, 20.9%): high initial sPAP followed by rapid decrease; trajectory 2 (n=44, 54.3%): moderate initial sPAP followed by slow decrease; trajectory 3 (n=13, 16.0%): low initial sPAP followed by slow decrease; trajectory 4 (n=7, 8.6%): high initial sPAP followed by rapid increase. The trajectories were identified using group-based trajectory modeling with cubic polynomial functions. Abbreviations: sPAP, systolic pulmonary artery pressure.

After establishing the four-group model, parameter estimates for different polynomial orders within each trajectory group are shown in **Supplementary Table 2**. The results demonstrated that when the polynomial order was set at three for each group, all p-values were <0.05; consequently, the final polynomial orders were determined as (3,3,3,3). When a five-group model with third-order polynomials was explored, the average posterior probabilities (APP) for each sPAP trajectory group during longitudinal follow-up were all >70% (**Table 2**), and the odds of correct classification (OCC) were all >5 (**Supplementary Table 3**), supporting the robustness of the classification. Based on these criteria, four distinct sPAP trajectories were identified (**Figure 1**): trajectory 1 (n = 17, 20.9%) characterized by high initial sPAP followed by a rapid decrease; trajectory 2 (n = 44, 54.3%) characterized by moderate initial sPAP followed by a slow decrease; trajectory 3 (n = 13, 16.0%) characterized by low initial sPAP followed by a slow decrease; and trajectory 4 (n = 7, 8.6%) characterized by high initial sPAP followed by a rapid increase. The APP of the four groups were 0.96, 0.98, 0.99, and 0.99, and the OCC were 11.85, 64.03, 251.76, and 232.13.

### Baseline clinical characteristics associated with each trajectory

**Table 3** shows the baseline characteristics that were significantly different among the four sPAP trajectory groups (all p < 0.05); the overall comparison of all characteristics is provided in **Supplementary Table 4**. Significant differences among the groups were observed for several key variables, including baseline pulmonary artery pressure, WHO functional class, 6−minute walking distance (6MWD), SLEDAI score, BNP level, pro−inflammatory cytokines (IL−6, IL−1β, and TNF−α), and glucocorticoid dose classification (all p < 0.05).

**Table 3:** Comparison of baseline characteristics across trajectory 4 groups in SLE-PAH cohort (p < 0.05).

Characteristics	Group 1 (N = 17)	Group 2 (N = 44)	Group 3 (N = 13)	Group 4 (N = 7)	P value
Baseline mPAP (mmHg)	75.71 (60.00, 87.00)	53.20 (46.00, 61.00)	38.69 (34.00, 43.00)	68.86 (52.50, 81.00)	<0.001
WHO functional class					<0.001
I	3 (17.6%)	12 (27.3%)	4 (30.8%)	0 (0.0%)	
II	4 (23.5%)	22 (50.0%)	3 (23.1%)	0 (0.0%)	
III	8 (47.1%)	8 (18.2%)	2 (15.4%)	1 (14.3%)	
IV	2 (11.8%)	2 (4.5%)	4 (30.8%)	6 (85.7%)	
6MWD (m)	39.71 (33.00, 45.80)	43.13 (38.85, 47.80)	42.46 (38.90, 48.40)	25.89 (25.30, 26.30)	<0.001
SLEDAI	5.12 (2.00, 6.00)	6.05 (4.00, 8.00)	5.92 (4.00, 8.00)	16.71 (14.50, 19.00)	<0.001
BNP (pg/mL)	357.32 (114.00, 511.00)	245.36 (66.88, 312.05)	185.34 (52.00, 200.00)	1848.20 (1582.50, 2153.66)	<0.001
IL-6	5.92 (2.10, 8.10)	7.49 (2.31, 8.34)	4.65 (2.38, 4.47)	97.06 (40.81, 104.19)	<0.001
IL-1β	5.73 (2.22, 6.82)	6.48 (2.75, 8.44)	5.66 (3.29, 7.68)	36.08 (27.52, 42.45)	<0.001
TNF-α	2.24 (2.04, 2.21)	2.28 (1.65, 2.66)	2.45 (2.05, 2.21)	12.99 (10.27, 14.55)	<0.001
Treatment					
Glucocorticoids dose classification					0.045
High dose	8 (44.4)	17 (42.5)	6 (40.0)	4 (50.0)	
Medium dose	5 (27.8)	16 (40.0)	5 (33.3)	3 (37.5)	
Low dose	5 (27.8)	5 (12.5)	4 (26.7)	0 (0.0)	

mPAP, mean pulmonary artery pressure; 6MWD, 6-minute walking distance; SLEDAI, Systemic Lupus Erythematosus Disease Activity Index; BNP, brain natriuretic peptide.

Patients in trajectory 4 (rapid progression, n = 7) exhibited the most severe phenotype at baseline: they had the highest median pulmonary artery pressure [68.86 mmHg (IQR 52.50–81.00)], the highest SLEDAI score [16.71 (14.50–19.00)], and the highest BNP level [1,848.20 pg/mL (1,582.50–2153.66)]. They also showed the poorest functional status, with 85.7% in WHO class IV and the shortest 6MWD distance [25.89 m (25.30–26.30)]. In contrast, patients in trajectory 3 (slow decrease from low baseline) had the mildest hemodynamic and clinical profile, including the lowest baseline mPAP [38.69 mmHg (34.00–43.00)] and the lowest SLEDAI score [5.92 (4.00–8.00)].

Notably, circulating levels of IL−6, IL−1β, and TNF−α were markedly elevated in trajectory 4 compared with the other three groups (all p < 0.001). For example, median IL−6 in trajectory 4 was 97.06 pg/mL (40.81–104.19) versus 4.65–7.49 pg/mL in the remaining groups. Glucocorticoid dose also differed significantly across groups (p = 0.045); trajectory 4 had the highest proportion of patients receiving high−dose glucocorticoids (50.0%) and none on low−dose therapy, consistent with its aggressive disease course.

### Multivariable analyses of factors associated with sPAP trajectory groups in SLE-PAH patients

**Figure 2:**
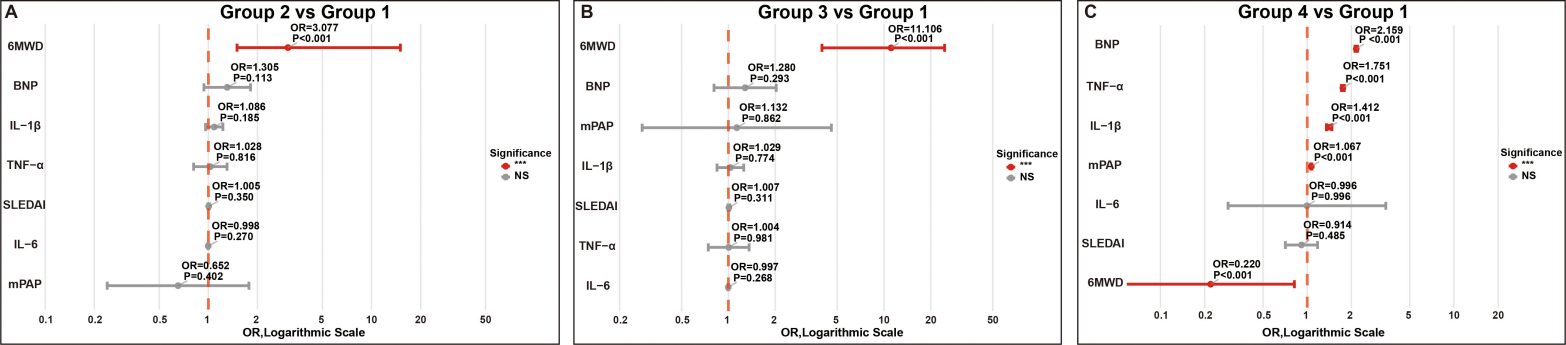
Multivariate analysis forest plot of related factors of sPAP trajectories in SLE-PAH patients. Forest plots showing ORs (95% CIs) on a logarithmic scale for trajectory membership vs. trajectory 1 (reference). The vertical dashed line indicates OR = 1. Reference group: trajectory 1; N = 81. Significance: ***P < 0.001, **P < 0.01, *P < 0.05, NS P ≥ 0.05. **(A)** Trajectory 2 vs. 1. Longer 6MWD was independently associated with trajectory 2 (OR = 3.077, P < 0.001). **(B)** Trajectory 3 vs. 1. Longer 6MWD was strongly associated with trajectory 3 (OR = 11.106, P < 0.001). **(C)** Trajectory 4 vs. 1. Shorter 6MWD (OR = 0.220), elevated BNP (OR = 2.159), IL-1β (OR = 1.412), TNF-α (OR = 1.751), and mPAP (OR = 1.067) were independently associated with trajectory 4 (all P < 0.001). 6MWD, 6-minute walking distance; BNP, brain natriuretic peptide; CI, confidence interval; IL-1β, interleukin-1β; IL-6, interleukin-6; mPAP, mean pulmonary artery pressure; OR, odds ratio; sPAP, systolic pulmonary artery pressure; SLE-PAH, systemic lupus erythematosus-associated pulmonary arterial hypertension; SLEDAI, Systemic Lupus Erythematosus Disease Activity Index; TNF-α, tumor necrosis factor-α.

To identify baseline characteristics independently associated with each sPAP trajectory, we performed multinomial logistic regression using trajectory 1 as the reference group. The results for comparisons with trajectory 2, trajectory 3, and trajectory 4 are summarized below (**Figures 2A-C**). 6MWD was the only independent predictor that significantly distinguished both trajectory 2 and trajectory 3 from trajectory 1, with ORs of 3.077 (95% CI 1.498–15.019) and 11.106 (95% CI 3.991–24.513), respectively (both p < 0.001). None of the other factors which include SLEDAI, BNP, IL−6, IL−1β, TNF−α, or mPAP showed a statistically significant association (all p > 0.05) (**Figures 2A, B**). In contrast, multiple baseline factors independently identified the rapid−progression trajectory. Shorter 6MWD was associated with a higher probability of belonging to trajectory 4 (OR = 0.220, 95% CI 0.059-0.819, p < 0.001). Elevated levels of BNP (OR = 2.159, 95% CI 2.117-2.202, p < 0.001), IL−1β (OR = 1.412, 95% CI 1.352-1.475, p < 0.001), TNF−α (OR = 1.751, 95% CI 1.712-1.791, p < 0.001), and mPAP (OR = 1.067, 95% CI 1.057–1.077, p < 0.001) each independently increased the odds of trajectory 4 membership. SLEDAI and IL−6 were not significant in this comparison (**Figure 2C**).

In summary, these findings highlight distinct baseline profiles: trajectories 2 and 3 are primarily differentiated by better functional capacity (longer 6MWD), whereas trajectory 4 is characterized by a combination of severe hemodynamic impairment, heightened pro−inflammatory cytokines (IL−1β and TNF−α), elevated BNP, and poor functional status. The lack of association with IL−6 in the multivariable model for trajectory 4 suggests that the inflammatory signature is more specific to IL−1β and TNF−α in this aggressive phenotype.

### Prognostic value of sPAP trajectories for mortality in SLE-PAH

**Figure 3:**
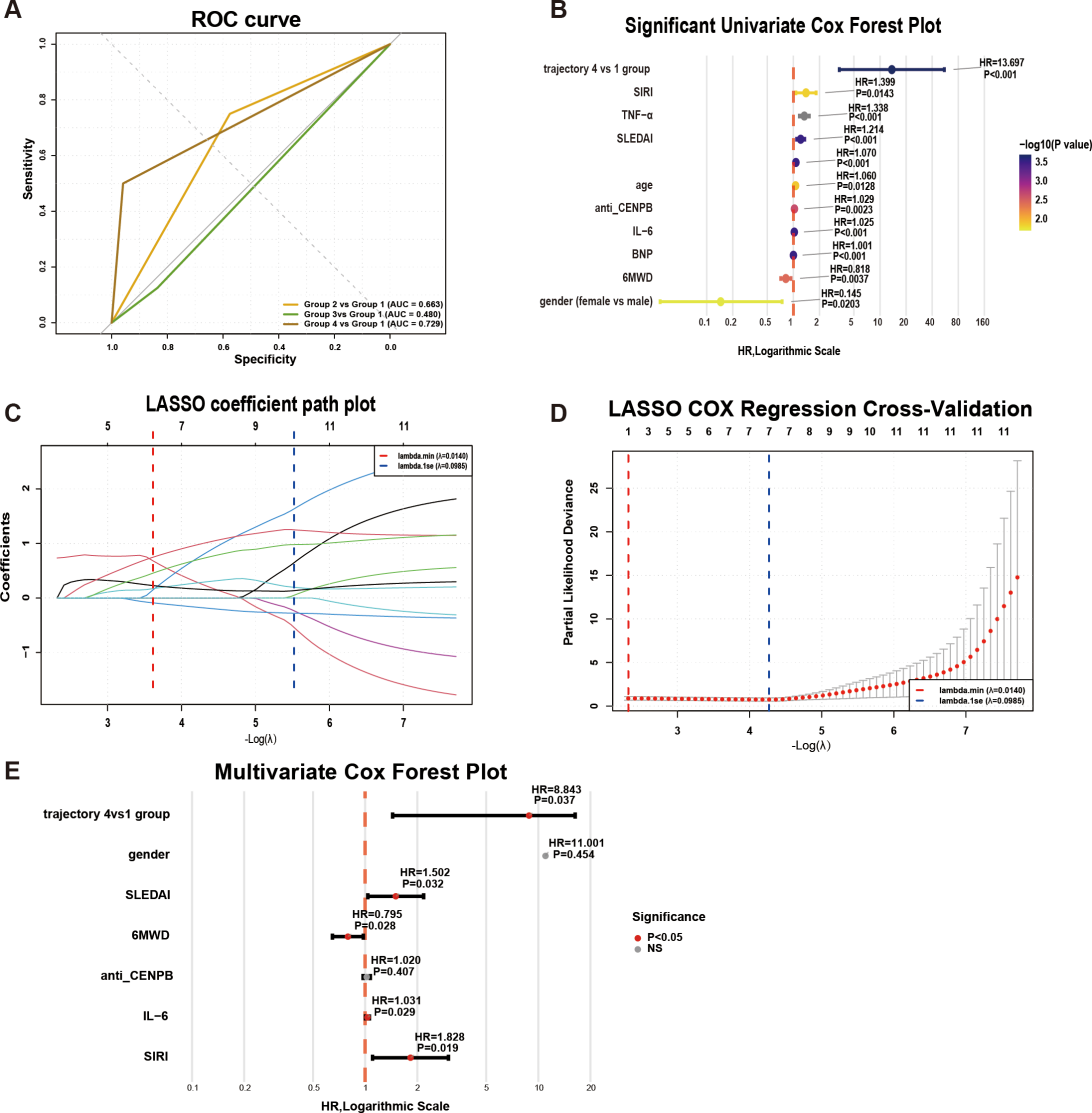
Prognostic value of sPAP trajectories and identification of independent mortality predictors in SLE-PAH patients. **(A)** Time-dependent ROC curves for mortality prediction. Trajectory 4 (rapid progression) demonstrated the highest predictive accuracy (AUC = 0.729) compared with trajectory 2 (AUC = 0.663) and trajectory 3 (AUC = 0.480), using trajectory 1 as reference. **(B)** Univariate Cox regression forest plot. Variables significantly associated with mortality (P < 0.05) included trajectory 4 (vs 1), SIRI, TNF-α, SLEDAI, age, anti-CENPB, IL-6, BNP, and 6MWD. Data are presented as hazard ratios (HR) with 95% confidence intervals on a logarithmic scale. **(C)** LASSO coefficient path plot. Coefficients of candidate predictors across different log(λ) values. The red vertical line indicates λ.min (λ = 0.0140), and the blue vertical line indicates λ.1se (λ = 0.0985). **(D)** LASSO Cox regression cross-validation plot. Partial likelihood deviance plotted against log(λ). **(E)** Multivariable Cox regression forest plot. Independent predictors of all-cause mortality after LASSO variable selection and multivariable adjustment. Trajectory 4 (HR = 8.843, P = 0.037), SLEDAI (HR = 1.502, P = 0.032), 6MWD (HR = 0.795, P = 0.028), IL-6 (HR = 1.031, P = 0.029), and SIRI (HR = 1.828, P = 0.019) remained significant. Gender and anti-CENPB were not statistically significant. 6MWD, 6-minute walking distance; anti-CENPB, anti-centromere protein B antibody; AUC, area under the curve; BNP, brain natriuretic peptide; CI, confidence interval; HR, hazard ratio; IL-6, interleukin-6; LASSO, least absolute shrinkage and selection operator; sPAP, systolic pulmonary artery pressure; ROC, receiver operating characteristic; SIRI, systemic inflammation response index; SLE-PAH, systemic lupus erythematosus-associated pulmonary arterial hypertension; SLEDAI, Systemic Lupus Erythematosus Disease Activity Index; TNF-α, tumor necrosis factor-α.

To evaluate the prognostic significance of the identified sPAP trajectories, we first assessed their predictive ability for all-cause mortality using time-dependent ROC curves (**Figure 3A**). Among the three pairwise comparisons with trajectory 1 (low stable) as reference, trajectory 4 (rapid progression) demonstrated the highest predictive accuracy, with an AUC of 0.729, whereas trajectory 2 vs 1 and trajectory 3 vs 1 yielded AUCs of 0.663 and 0.480, respectively. This indicated that the rapid−progression trajectory (trajectory 4) was the most informative for mortality risk stratification. Univariate Cox regression analysis was then performed to screen potential prognostic factors. As shown in **Figure 3B**, **Supplementary Table 5**, 11 variables were significantly associated with mortality (p < 0.05): trajectory 4 vs 1, SIRI, TNF−α, SLEDAI, IL−1β, age, anti−CENPB, IL−6, BNP, 6MWD, and gender. Detailed univariate results are provided in **Supplementary Table 5**. To further refine variable selection and avoid overfitting, we applied LASSO regression with 10−fold cross−validation (**Figures 3C, D**). The optimal penalty parameter λ was determined at λ·min = 0.0140 and λ.1se = 0.0985. Variables with non−zero coefficients at λ·min were retained for subsequent multivariable analysis.

**Figure 4:**
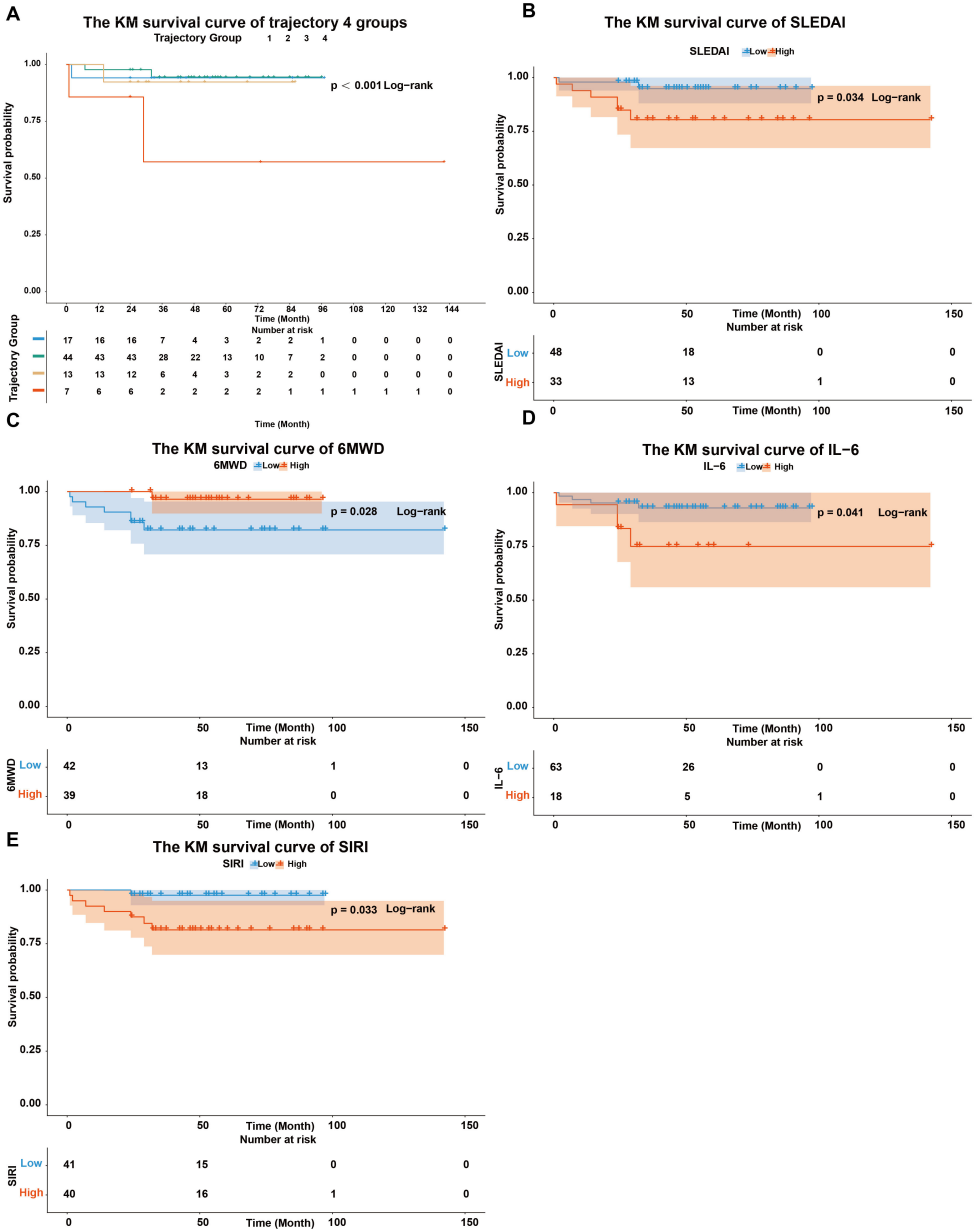
Kaplan–Meier survival curves for independent prognostic factors in SLE-PAH patients. **(A)** The KM survival curve of Trajectory 4 group. Patients in trajectory 4 (rapid progression) exhibited significantly worse survival compared with trajectories 1–3 (log-rank P < 0.001). **(B)** The KM survival curve of the SLEDAI 4 group. Patients with higher SLEDAI (≥median) had significantly poorer survival than those with lower SLEDAI (log-rank P = 0.034). **(C)** The KM survival curve of the 6MWD group. Patients with shorter 6MWD (≤median) demonstrated significantly worse survival compared with those with longer 6MWD (log-rank P = 0.028). **(D)** The KM survival curve of the IL-6 group. Patients with elevated IL-6 (≥median) had significantly reduced survival compared with those with lower IL-6 (log-rank P = 0.041). **(E)** The KM survival curve of the SIRI group. Patients with higher SIRI (≥median) experienced significantly worse survival than those with lower SIRI (log-rank P = 0.033). 6MWD, 6-minute walking distance; IL-6, interleukin-6; sPAP, systolic pulmonary artery pressure; SIRI, systemic inflammation response index; SLE-PAH, systemic lupus erythematosus-associated pulmonary arterial hypertension; SLEDAI, Systemic Lupus Erythematosus Disease Activity Index.

Multivariable Cox regression incorporating the LASSO−selected variables identified five independent predictors of mortality (**Figure 3E**, **Supplementary Table 6**). After adjustment, trajectory 4 remained a strong independent risk factor (HR = 8.843, 95% CI 1.441-16.314, p = 0.037). Other significant factors included higher SLEDAI (HR = 1.502, 95% CI 1.036-2.177, p = 0.032), elevated IL−6 (HR = 1.031, 95% CI 1.003–1.059, p = 0.029), higher SIRI (HR = 1.828, 95% CI 1.104–3.026, p = 0.019), and shorter 6MWD (HR = 0.795, 95% CI 0.647-0.976, p = 0.028). Gender and anti−CENPB did not retain statistical significance in the multivariable model. Finally, Kaplan–Meier survival curves were constructed for each independent predictor, with continuous variables dichotomized at the median (**Figure 4**). Patients classified into trajectory 4, those with higher SLEDAI, elevated IL−6, higher SIRI, or shorter 6MWD exhibited significantly worse survival (all log−rank p < 0.05). These findings underscore the robust prognostic value of the sPAP trajectory classification, particularly the rapid−progression phenotype, in predicting mortality among SLE−PAH patients.

## Discussion

In this study, we employed latent trajectory modeling to delineate the temporal evolution of sPAP in patients with systemic lupus erythematosus−associated pulmonary arterial hypertension (SLE−PAH) and identified four distinct trajectories with unique patterns of sPAP change. These trajectories differed not only in baseline clinical characteristics but also in long−term prognosis. Specifically, trajectory 4, which had an initially high sPAP value and then rapidly increased, exhibited the most aggressive phenotype and had significantly poorer survival rates compared with the other groups. In contrast, trajectories 2 (moderate initial sPAP with slow decrease) and 3 (low initial sPAP with slow decrease) were associated with better functional capacity (longer 6−minute walking distance) and a more favorable outcome, whereas trajectory 1 (high initial sPAP followed by a rapid decrease) occupied an intermediate position. Notably, over half of the patients (54.3%) belonged to trajectory 2, indicating that a moderate−onset, slow−decline pattern represents the most common clinical course in this cohort.

6MWD is a powerful independent factor distinguishing distinct sPAP trajectories in patients with SLE-PAH. Compared with trajectory 1 (high initial sPAP with rapid decrease), longer 6MWD was independently associated with both trajectory 2 (moderate initial sPAP with slow decrease; OR = 3.077, p < 0.001) and trajectory 3 (low initial sPAP with slow decrease; OR = 11.106, p < 0.001), despite shorter 6MWD independently predicted trajectory 4 membership (rapid progression; OR = 0.220, p < 0.001). In PAH, exercise capacity as measured by the 6MWD is widely recognized as an integrated functional endpoint reflecting hemodynamic severity and right ventricular performance (10). Multiple studies have demonstrated that elevated pulmonary arterial pressures and increased pulmonary vascular resistance, hallmarks of PAH pathophysiology, are associated with reduced exercise tolerance and shorter 6MWD, indicating a negative correlation between mean pulmonary arterial pressure (mPAP) and 6MWD in PAH populations (18–20). Invasive assessments have shown that higher mPAP and pulmonary vascular resistance correlates with poorer 6MWD outcomes, supporting the link between hemodynamic impairment and functional limitation in PAH patients (21, 22).

Furthermore, our survival analysis demonstrated that 6MWD serves as a powerful predictor of prognosis in SLE-PAH. In the multivariable Cox regression model, shorter 6MWD remained an independent risk factor for all-cause mortality (HR = 0.795, 95% CI 0.647–0.976, p = 0.028), even after adjustment for hemodynamic parameters, inflammatory markers, and lupus disease activity. Kaplan–Meier analysis further confirmed that patients with 6MWD below the median experienced significantly worse survival (log-rank p = 0.001). These findings position 6MWD as a robust and clinically accessible prognostic marker in SLE-PAH. Our results are highly consistent with the landmark multicenter prospective cohort study from CSTAR-PAH, which enrolled 310 patients from 14 PAH centers across China (23). A CSTAR−derived prognostic model identified 6MWD among eight core predictors (C−index 0.77) (24). Zhao et al. reported that 6MWD ≤380 m conferred a >3−fold mortality risk in CTD−PAH (HR = 3.222) (25); Guo et al. showed that combining 6MWD with the pulmonary−to−systemic vascular resistance ratio improved predictive accuracy (C−index 0.75) (26). A recent European cohort study from Austria evaluated the prognostic utility of functional capacity assessment in 114 patients with pulmonary hypertension (mean age 66 ± 14 years). The study established that 6MWD thresholds of <165 and >440 m, as recommended by ESC/ERS guidelines, effectively discriminated 1-year mortality risk (27). These consistent findings across various cohorts (from a single center to large registry studies) have established the 6-minute walk distance (6MWD) as a universally applicable prognostic cornerstone. The objective, simple, and non-invasive nature of 6MWD, combined with its strong correlation with hard clinical outcomes across diverse populations, positions it as an indispensable tool for risk stratification and longitudinal monitoring in routine clinical practice.

The independent predictive value of baseline mean pulmonary artery pressure (mPAP) for trajectory 4 membership further underscores the importance of initial hemodynamic severity in determining disease progression, which can influence the trajectory of sPAP. Similarly, in moderate rheumatic mitral stenosis, baseline PASP >40 mmHg is an independent predictor of the “rapid PASP progression” trajectory, which is associated with a higher risk of death or hospitalization for heart failure (28). However, baseline mPAP differed significantly across trajectory groups in univariate analysis; it did not emerge as an independent predictor of mortality in the multivariable Cox regression model. Instead, trajectory 4 membership characterized by high initial sPAP followed by rapid progression remained a strong independent risk factor for death (HR = 8.843, 95% CI 1.441–16.314, p = 0.037). This finding underscores a critical insight: A single baseline hemodynamic measurement may be insufficient to capture the full prognostic implications of disease progression in SLE-PAH. This further indicates that although baseline mPAP serves as a primary analytical variable in predicting SLE-PAH prognosis, its predictive capability does not operate in isolation (29).

The superiority of longitudinal trajectory analysis over static baseline measurements aligns with emerging evidence in the field of pulmonary hypertension research. A recent study employing a cohort-based trajectory model analyzed the longitudinal patterns of mean pulmonary artery wedge pressure (mPAWP) changes over a 10-year period in 301 patients with pulmonary arterial hypertension (PAH). The research results show that although an initial mean pulmonary artery pressure (mPAWP) of ≥12 mmHg significantly predicts that patients will enter a high mPAWP trajectory (odds ratio = 3.2, p = 0.0006), the factor that is truly significantly associated with the shortened pre-transplant survival period is the trajectory itself (rather than the simple baseline value). The median survival periods of the two groups were 7.8 and 11.3 years, respectively (log-rank test p = 0.017) (8). This demonstrates that longitudinal trajectories outperform single time-point measurements in prognostic discrimination directly, which parallels our findings in SLE-PAH. Kida et al. applied latent trajectory modeling to sPAP changes in 236 patients with systemic sclerosis (SSc) and identified five distinct trajectories of PAP elevation. Consistent with our research findings, these trajectories (arranged in the order of the increase in sPAP over time) indicate a correspondingly shorter survival period without pulmonary hypertension. Crucially, despite that baseline clinical characteristics were associated with trajectory assignment, it was the temporal pattern of sPAP change that provided the most clinically meaningful stratification for long-term outcomes (9). These findings, together with our own, suggest that the dynamic evolution of pulmonary hemodynamics captures pathophysiological processes, such as progressive vascular remodeling, right ventricular maladaptation, and accelerating inflammation.

Patients in trajectory 4 exhibited significantly elevated baseline levels of BNP (OR = 2.159, p < 0.001), IL−1β (OR = 1.412, p < 0.001), TNF−α (OR = 1.751, p < 0.001), and mean pulmonary artery pressure (mPAP) (OR = 1.067, p < 0.001). These findings delineate a high-risk phenotype characterized by the convergence of hemodynamic severity, right ventricular strain, and systemic inflammation. A growing body of evidence supports the concept that inflammation and hemodynamic forces are not independent processes but rather engage in a bidirectional, mutually reinforcing relationship in pulmonary arterial hypertension. Liu et al. proposed that altered hemodynamic forces cause endothelial dysfunction, leading to immune cell adherence and release of inflammatory mediators; the resulting perivascular inflammation, in turn, promotes vascular remodeling and disease progression, creating a “vicious cycle of endothelial activation, inflammation, and vascular remodeling” that drives the disease process (30). The mechanistic basis for this phenomenon likely lies in the complex interplay between hemodynamic load, ventricular adaptation, and systemic inflammation over time (31, 32). A single elevated mPAP measurement may identify patients with established hemodynamic compromise, but it cannot distinguish those whose disease will remain stable from those destined for rapid deterioration. In contrast, the trajectory of sPAP evolution integrates information about the rate of disease progression, the adequacy of compensatory mechanisms, and the cumulative impact of inflammatory and vascular injury (33). The above results indicated that trajectory 4 represented the most aggressive phenotype, associated with the highest mortality. Patients in this trajectory exhibited significantly elevated baseline levels of BNP, IL−1β, TNF−α, and mPAP, as well as the shortest 6−minute walk distance (all p < 0.05). These results also suggest that patients in trajectory 4 should have echocardiography, BNP, and 6MWD rechecked every 3 months in the first year and at least every 6 months thereafter. In terms of treatment, it is recommended that patients in trajectory 4 be initiated on combined immunosuppressive therapy early to control lupus activity and start dual PAH-targeted therapy simultaneously, rather than sequential monotherapy. For patients in trajectories 2 and 3, single-drug PAH treatment or conventional immunosuppressive therapy can be used initially. Only when there are signs of a shift to a high-risk trajectory, such as a decrease in 6MWD or an increase in BNP, should the treatment be upgraded.

Multivariable Cox regression analysis identified several independent predictors of all-cause mortality in SLE-PAH patients. In addition to trajectory 4 membership, higher SLEDAI score (HR = 1.502, p = 0.032), elevated IL-6 levels (HR = 1.031, p = 0.029), higher SIRI (HR = 1.828, p = 0.019), and shorter 6MWD (HR = 0.795, p = 0.028) emerged as significant prognostic factors. These findings underscore the multifactorial nature of mortality risk in SLE-PAH, encompassing lupus disease activity, systemic inflammation, functional capacity, and hemodynamic trajectory. The finding that higher IL−6 and SIRI associate with mortality aligns with broader evidence that systemic inflammation contributes both to SLE−PAH pathogenesis and to worse survival in inflammatory and cardiopulmonary diseases (34–36) In our cohort, a higher SLEDAI score was an independent predictor of mortality, which is consistent with the results from previous studies showing that “high disease activity significantly increases the risk of death” in patients with SLE and SLE-PAH. This highlights the crucial role of controlling systemic diseases in the prognosis of SLE-PAH (37, 38).

This study has several limitations. Firstly, although derived from the prospective CSTAR-PAH cohort, our analysis was retrospective in nature. Notably, the available data were not obtained through standardized prospective clinical assessments, which resulted in some missing information. We employed the multiple imputation method, which is a widely accepted approach for handling missing data to solve this problem. However, the mechanisms underlying the missingness patterns may be complex and could potentially introduce bias into the estimates. Future studies with more complete prospective data collection are warranted to obtain more precise estimates. Secondly, although echocardiography screening was conducted every 3 months during the first year of follow-up, the schedule for assessment after the first year was not standardized. This lack of uniformity in screening intervals during the later follow-up period may have influenced the identification of trajectory group differences, particularly for trajectories characterized by later divergence. Prospective studies with fixed, long-term echocardiographic monitoring schedules are needed to validate our trajectory classification. Additionally, the proportion of patients in the trajectory 4 is low in our single-center cohort, and future multicenter studies with larger sample sizes are needed to further validate the stability and clinical significance of this trajectory.

## Conclusion

In conclusion, our study demonstrates that sPAP trajectories capture clinically meaningful heterogeneity in SLE−PAH and provide prognostic discrimination superior to baseline mPAP alone. The rapid progression trajectory (trajectory 4) identified a high-risk subgroup characterized by significant inflammatory responses (increased levels of IL-1β, TNF-α, IL-6, and SIRI), right ventricular strain (increased BNP), hemodynamic impairment (increased mean pulmonary artery pressure), and functional impairment (shortened 6-minute walking test time). These factors, independent of traditional risk factors, significantly increased the risk of death (HR = 8.843, p = 0.037). In addition to the disease course trajectory, higher SLEDAI scores, elevated IL-6 levels, higher SIRI, and shorter 6MWD have all been proven to be independent predictors of death. This highlights the multifactorial nature of the risk of death, including autoimmune activity, systemic inflammation, functional status, and hemodynamic trajectory. These findings support the use of trajectory−based phenotyping for risk stratification and personalized management in SLE−PAH.

## Data Availability

The raw data supporting the conclusions of this article will be made available by the authors, without undue reservation.
