# Supplementary material for: Implications of systolic pulmonary artery pressure trajectories in systemic lupus erythematosus-associated pulmonary hypertension: a CSTAR-PAH cohort study
Source: Front Immunol. 2026 Jun 9;17:1819805. doi: 10.3389/fimmu.2026.1819805 (PMC13286918; doi:10.3389/fimmu.2026.1819805)
Supplement: Supplementary file 1 [file Table1.docx]

# **Supplementary Table 1. Missing data summary before multiple imputation.**

| **Characteristics** | **Missing count** | **Missing percent** |
| --- | --- | --- |
| **Essential characteristic** | | |
| Age (m) | 0 | 0% |
| Gender | 0 | 0% |
| Disease duration (m) | 0 | 0% |
| BMI | 0 | 0% |
| Trajectory 4 group | 0 | 0% |
| Death | 0 | 0% |
| Baseline mPAP (mmHg) | 0 | 0% |
| **Risk stratification parameters** | | |
| WHO functional class | 0 | 0% |
| 6MWD (m) | 0 | 0% |
| SLEDAI | 0 | 0% |
| **Clinical feature** | | |
| Acute subacute cutaneous lupus | 0 | 0% |
| Chronic cutaneous lupus | 0 | 0% |
| Oral ulcers | 0 | 0% |
| Non scarring alopecia | 0 | 0% |
| Arthritis | 0 | 0% |
| Hemolytic anemia | 0 | 0% |
| Leukopenia | 0 | 0% |
| Thrombocytopenia | 0 | 0% |
| TTP | 0 | 0% |
| Serositis | 0 | 0% |
| Lupus nephritis | 0 | 0% |
| Interstitial lung disease | 0 | 0% |
| Gastrointestinal | 0 | 0% |
| Neuropathy | 0 | 0% |
| ****Laboratory parameters**** | | |
| WBC (×10⁹/L) | 0 | 0% |
| Hb (g/L) | 0 | 0% |
| PLT (×10⁹/L) | 0 | 0% |
| Neutrophil count (×10⁹/L) | 0 | 0% |
| Lymphocyte count (×10⁹/L) | 0 | 0% |
| Monocyte count (×10⁹/L) | 0 | 0% |
| Albumin (g/L) | 0 | 0% |
| Total bilirubin (μmol/L) | 0 | 0% |
| Direct bilirubin (μmol/L) | 0 | 0% |
| AST (U/L) | 0 | 0% |
| ALT (U/L) | 0 | 0% |
| LDH (U/L) | 0 | 0% |
| Uric acid (μmol/L) | 0 | 0% |
| Creatinine (μmol/L) | 0 | 0% |
| eGFR (mL/min/1.73m²) | 0 | 0% |
| BNP (pg/mL) | 0 | 0% |
| **Inflammatory variables** | | |
| ESR (mm/h) | 1 | 1.23% |
| CRP (mg/L) | 1 | 1.23% |
| SII | 0 | 0% |
| SIRI | 0 | 0% |
| NLR | 0 | 0% |
| **Serology and autoantibodies** | | |
| IgG (g/L) | 1 | 1.23% |
| IgA (g/L) | 1 | 1.23% |
| IgM (g/L) | 1 | 1.23% |
| C3 (g/L) | 1 | 1.23% |
| C4 (g/L) | 1 | 1.23% |
| ANA titer | 1 | 1.23% |
| Anti dsDNA (IU/mL) | 1 | 1.23% |
| ANA C1q | 4 | 4.94% |
| Anti Sm | 4 | 4.94% |
| Anti nRNP | 4 | 4.94% |
| Anti SSA | 4 | 4.94% |
| Anti Ro52 | 4 | 4.94% |
| Anti SSB | 4 | 4.94% |
| Anti Scl70 | 4 | 4.94% |
| Anti PM Scl | 4 | 4.94% |
| Anti Jo1 | 4 | 4.94% |
| Anti ANUA | 4 | 4.94% |
| Anti CENPB | 4 | 4.94% |
| Anti histone | 4 | 4.94% |
| Anti RIB P | 4 | 4.94% |
| Anti AMA M2 | 4 | 4.94% |
| Lupus anticoagulant ratio | 0 | 0% |
| aCL IgA | 8 | 9.88% |
| aCL IgG | 8 | 9.88% |
| aCL IgM | 8 | 9.88% |
| Anti β2 glycoprotein IgA | 8 | 9.88% |
| Anti β2 glycoprotein IgG | 8 | 9.88% |
| Anti β2 glycoprotein IgM | 8 | 9.88% |
| **Lymphocyte subsets** | | |
| T cell percent | 5 | 6.17% |
| T helper percent | 5 | 6.17% |
| T suppressor percent | 5 | 6.17% |
| NK cell percent | 5 | 6.17% |
| B cell percent | 5 | 6.17% |
| DPT cell percent | 8 | 9.88% |
| DNT cell percent | 8 | 9.88% |
| NK T cell percent | 8 | 9.88% |
| T cell count | 8 | 9.88% |
| T helper count | 8 | 9.88% |
| T suppressor count | 8 | 9.88% |
| NK cell count | 8 | 9.88% |
| B cell count | 8 | 9.88% |
| DPT cell count | 8 | 9.88% |
| DNT cell count | 8 | 9.88% |
| NKT cell count | 8 | 9.88% |
| TBNK percent | 8 | 9.88% |
| TBNK count | 8 | 9.88% |
| THTS ratio | 8 | 9.88% |
| **Cytokines **(pg/mL)**** | | |
| IL-5 | 4 | 4.94% |
| IFN-α | 4 | 4.94% |
| IL-2 | 4 | 4.94% |
| IL-6 | 4 | 4.94% |
| IL-1β | 4 | 4.94% |
| IL-10 | 4 | 4.94% |
| IFN-γ | 4 | 4.94% |
| IL-8 | 4 | 4.94% |
| IL-17 | 4 | 4.94% |
| IL-4 | 4 | 4.94% |
| IL-12p70 | 4 | 4.94% |
| TNF-α | 4 | 4.94% |
| **Pulmonary function tests** | | |
| FVC percent | 8 | 9.88% |
| FEV1 | 8 | 9.88% |
| TLC | 8 | 9.88% |
| DLCO | 8 | 9.88% |
| **Treatment** | | |
| Glucocorticoids | 0 | 0% |
| Hydroxychloroquine sulfate | 0 | 0% |
| Cyclophosphamide | 0 | 0% |
| Mycophenolate mofetil | 0 | 0% |
| Ciclosporin A | 0 | 0% |
| Tacrolimus | 0 | 0% |
| Azathioprine | 0 | 0% |
| Methotrexate | 0 | 0% |
| Iguratimod | 0 | 0% |
| Endothelin receptor antagonist | 0 | 0% |
| Phosphodiesterase 5 inhibitor | 0 | 0% |
| Prostaglandin analogs | 0 | 0% |

Abbreviations: BMI, body mass index; mPAP, mean pulmonary artery pressure; 6MWD, 6-minute walking distance; SLEDAI, Systemic Lupus Erythematosus Disease Activity Index; TTP, thrombotic thrombocytopenic purpura; ILD, interstitial lung disease; WBC, white blood cell count; Hb, hemoglobin; PLT, platelet; AST, aspartate aminotransferase; ALT, alanine aminotransferase; LDH, lactate dehydrogenase; eGFR, estimated glomerular filtration rate; BNP, brain natriuretic peptide; ESR, erythrocyte sedimentation rate; CRP, C-reactive protein; SII, systemic immune-inflammation index; SIRI, systemic inflammation response index; NLR, neutrophil-to-lymphocyte ratio; Ig, immunoglobulin; C3, complement 3; C4, complement 4; ANA, antinuclear antibody; anti-dsDNA, anti-double-stranded DNA antibody; anti-Sm, anti-Smith antibody; anti-nRNP, anti-ribonucleoprotein antibody; anti-SSA, anti-Sjögren's syndrome antigen A antibody; anti-Ro52, anti-Ro52 antibody; anti-SSB, anti-Sjögren's syndrome antigen B antibody; anti-Scl70, anti-topoisomerase I antibody; anti-PM-Scl, anti-polymyositis-scleroderma antibody; anti-Jo1, anti-histidyl-tRNA synthetase antibody; anti-CENPB, anti-centromere protein B antibody; anti-ANUA, anti-nucleosome antibody; anti-RIB P, anti-ribosomal P protein antibody; anti-AMA-M2, anti-mitochondrial antibody M2; aCL, anticardiolipin antibody; anti-β2GPI, anti-β2 glycoprotein I antibody; NK cell, natural killer cell; DPT cell, double-positive T cell; DNT cell, double-negative T cell; NKT cell, natural killer T cell; TBNK, T cells, B cells, and NK cells; THTS, T helper/T suppressor ratio; IL, interleukin; IFN, interferon; TNF, tumor necrosis factor; FVC, forced vital capacity; FEV1, forced expiratory volume in 1 second; TLC, total lung capacity; DLCO, diffusing capacity of the lung for carbon monoxide; MMF, mycophenolate mofetil; ERA, endothelin receptor antagonist; PDE5i, phosphodiesterase type 5 inhibitor.

**Supplementary Table 2. Parameter estimates for different polynomial orders within each trajectory group .**

| **Group** | **Parameter** | **Estimate** | **Std. Error** | **t value** | **P value** |
| --- | --- | --- | --- | --- | --- |
| 1 | Intercept | 80.165347 | 1.879768 | 42.646 | <0.0001 |
| 1 | I(t^1) | -7.533222 | 0.893993 | -8.426 | <0.0001 |
| 1 | I(t^2) | 0.556801 | 0.094371 | 5.900 | <0.0001 |
| 1 | I(t^3) | -0.012806 | 0.002502 | -5.119 | <0.0001 |
| 2 | Intercept | 57.039306 | 1.273368 | 44.794 | <0.0001 |
| 2 | I(t^1) | -5.493880 | 0.605597 | -9.072 | <0.0001 |
| 2 | I(t^2) | 0.405506 | 0.063927 | 6.343 | <0.0001 |
| 2 | I(t^3) | -0.009114 | 0.001695 | -5.378 | <0.0001 |
| 3 | Intercept | 41.965032 | 0.707324 | 59.329 | <0.0001 |
| 3 | I(t^1) | -5.182080 | 0.336394 | -15.405 | <0.0001 |
| 3 | I(t^2) | 0.400588 | 0.035510 | 11.281 | <0.0001 |
| 3 | I(t^3) | -0.009214 | 0.000941 | -9.788 | <0.0001 |
| 4 | Intercept | 69.650399 | 2.214281 | 31.455 | <0.0001 |
| 4 | I(t^1) | 6.745419 | 1.053083 | 6.405 | <0.0001 |
| 4 | I(t^2) | -0.533960 | 0.111164 | -4.803 | <0.0001 |
| 4 | I(t^3) | 0.012414 | 0.002947 | 4.213 | <0.0001 |

Abbreviations: Est., Estimate; SE, Standard Error; t, t value; P, P value.

**Supplementary Table 3. OCC for each trajectory group in the four-group model.**

| **Model** | **OCC** | | | |
| --- | --- | --- | --- | --- |
|  | **G1** | **G2** | **G3** | **G4** |
| traj_4 | 11.854 | 64.034 | 251.764 | 232.133 |

Abbreviations: traj_4，trajectory model 4；OCC，odds of correct classification.

### ****Supplementary Table 4. Comparison of Baseline Characteristics Across Trajectory 4 Groups in SLE-PAH Cohort****

| **Characteristics** | **Group 1**  **(N = 17)** | **Group 2**  **(N = 44)** | **Group 3**  **(N = 13)** | **Group 4**  **(N = 7)** | **P**  **value** |
| --- | --- | --- | --- | --- | --- |
| ****Essential characteristic**** | | | | | |
| Age (m) | 46.00 (38.00, 53.00) | 45.00 (37.00, 53.25) | 38.00 (37.00, 55.00) | 50.00 (32.00, 54.50) | 0.948 |
| Gender |  |  |  |  | 0.434 |
| Male | 2 (11.8%) | 2 (4.5%) | 1 (7.7%) | 1 (14.3%) |  |
| Female | 15 (88.2%) | 42 (95.5%) | 12 (92.3%) | 6 (85.7%) |  |
| Disease duration (m) | 40.71 (24.00, 45.00) | 49.80 (24.00, 65.25) | 43.15 (27.00, 52.00) | 45.29 (24.00, 51.00) | 0.376 |
| BMI | 20.70 (18.20, 23.83) | 19.90 (18.06, 21.24) | 21.46 (18.82, 24.34) | 19.00 (17.65, 19.56) | 0.271 |
| Death | 1 (5.9%) | 2 (4.5%) | 1 (7.7%) | 2 (28.6%) | 0.144 |
| Baseline mPAP (mmHg) | 75.71 (60.00, 87.00) | 53.20 (46.00, 61.00) | 38.69 (34.00, 43.00) | 68.86 (52.50, 81.00) | <0.001 |
| ****Risk stratification parameters**** | | | | | |
| WHO functional class |  |  |  |  | <0.001 |
| I | 3 (17.6%) | 12 (27.3%) | 4 (30.8%) | 0 (0.0%) |  |
| II | 4 (23.5%) | 22 (50.0%) | 3 (23.1%) | 0 (0.0%) |  |
| III | 8 (47.1%) | 8 (18.2%) | 2 (15.4%) | 1 (14.3%) |  |
| IV | 2 (11.8%) | 2 (4.5%) | 4 (30.8%) | 6 (85.7%) |  |
| 6MWD (m) | 39.71 (33.00, 45.80) | 43.13 (38.85, 47.80) | 42.46 (38.90, 48.40) | 25.89 (25.30, 26.30) | <0.001 |
| SLEDAI | 5.12 (2.00, 6.00) | 6.05 (4.00, 8.00) | 5.92 (4.00, 8.00) | 16.71 (14.50, 19.00) | <0.001 |
| ****Clinical feature**** | | | | | |
| Acute/subacute cutaneous lupus | 8 (47.1%) | 9 (20.5%) | 3 (23.1%) | 0 (0.0%) | 0.075 |
| Chronic cutaneous lupus | 2 (11.8%) | 3 (6.8%) | 3 (23.1%) | 0 (0.0%) | 0.296 |
| Oral ulcers | 2 (11.8%) | 0 (0.0%) | 1 (7.7%) | 0 (0.0%) | 0.102 |
| Non-scarring alopecia | 1 (5.9%) | 9 (20.5%) | 2 (15.4%) | 2 (28.6%) | 0.429 |
| Arthritis | 9 (52.9%) | 12 (27.3%) | 3 (23.1%) | 3 (42.9%) | 0.217 |
| Hemolytic anemia | 1 (5.9%) | 6 (13.6%) | 1 (7.7%) | 0 (0.0%) | 0.836 |
| Leukopenia | 6 (35.3%) | 15 (34.1%) | 3 (23.1%) | 5 (71.4%) | 0.218 |
| Thrombocytopenia | 9 (52.9%) | 16 (36.4%) | 4 (30.8%) | 4 (57.1%) | 0.469 |
| TTP | 1 (5.9%) | 0 (0.0%) | 0 (0.0%) | 0 (0.0%) | 0.457 |
| Serositis | 4 (23.5%) | 14 (31.8%) | 5 (38.5%) | 3 (42.9%) | 0.723 |
| Lupus nephritis | 3 (17.6%) | 15 (34.1%) | 3 (23.1%) | 2 (28.6%) | 0.647 |
| Interstitial lung disease | 3 (17.6%) | 6 (13.6%) | 2 (15.4%) | 0 (0.0%) | 0.815 |
| Gastrointestinal | 0 (0.0%) | 1 (2.3%) | 1 (7.7%) | 0 (0.0%) | 0.477 |
| Neuropathy | 2 (11.8%) | 2 (4.5%) | 0 (0.0%) | 1 (14.3%) | 0.257 |
| ****Laboratory parameters**** | | | | | |
| WBC (×10⁹/L) | 6.17 (3.89, 7.99) | 6.08 (3.52, 7.19) | 6.28 (3.88, 7.44) | 5.21 (3.12, 7.18) | 0.942 |
| Hemoglobin (g/L) | 116.82 (94.00, 134.00) | 103.11 (89.50, 122.00) | 107.08 (94.00, 117.00) | 107.04 (105.00, 139.00) | 0.354 |
| Platelets (×10⁹/L) | 170.94 (107.00, 225.00) | 179.27 (117.00, 223.50) | 204.46 (135.00, 249.00) | 153.14 (108.50, 190.50) | 0.834 |
| Neutrophil count (×10⁹/L) | 4.95 (2.40, 7.69) | 4.24 (2.20, 6.30) | 4.78 (2.83, 6.31) | 3.17 (2.04, 3.66) | 0.480 |
| Lymphocyte count (×10⁹/L) | 1.34 (0.59, 1.50) | 1.44 (0.75, 1.84) | 1.36 (0.81, 1.76) | 1.28 (0.66, 1.46) | 0.774 |
| Monocyte count (×10⁹/L) | 0.33 (0.08, 0.47) | 0.45 (0.10, 0.74) | 0.22 (0.05, 0.30) | 0.67 (0.36, 0.85) | 0.077 |
| Albumin (g/L) | 35.46 (31.43, 39.92) | 34.98 (29.72, 40.03) | 66.99 (31.48, 41.23) | 31.78 (29.60, 33.02) | 0.467 |
| Total bilirubin (μmol/L) | 12.37 (8.20, 14.89) | 20.74 (7.26, 13.09) | 10.04 (7.20, 10.31) | 9.12 (6.15, 10.88) | 0.224 |
| Direct bilirubin (μmol/L) | 3.84 (1.69, 4.98) | 8.76 (1.57, 3.91) | 2.50 (1.60, 2.80) | 3.10 (1.25, 3.59) | 0.723 |
| AST (U/L) | 28.83 (19.40, 32.84) | 40.37 (19.40, 36.91) | 26.54 (21.30, 28.85) | 37.41 (20.59, 35.00) | 0.883 |
| ALT (U/L) | 19.89 (11.05, 24.88) | 28.63 (10.60, 26.52) | 20.28 (10.73, 18.48) | 34.76 (10.35, 39.93) | 0.977 |
| LDH (U/L) | 121.78 (2.06, 257.20) | 116.65 (2.10, 185.52) | 91.90 (2.44, 188.46) | 145.02 (2.95, 245.94) | 0.723 |
| Uric acid (μmol/L) | 171.79 (4.22, 350.45) | 151.02 (4.41, 341.52) | 100.60 (5.28, 197.93) | 240.03 (11.52, 420.55) | 0.405 |
| Creatinine (μmol/L) | 87.64 (52.80, 72.66) | 82.78 (50.82, 72.79) | 64.42 (45.95, 63.52) | 76.68 (68.22, 83.37) | 0.225 |
| eGFR (mL/min/1.73m²) | 100.91 (79.93, 111.47) | 105.00 (80.94, 120.98) | 108.33 (93.77, 139.42) | 88.45 (69.53, 92.46) | 0.359 |
| BNP (pg/mL) | 357.32 (114.00, 511.00) | 245.36 (66.88, 312.05) | 185.34 (52.00, 200.00) | 1848.20 (1582.50, 2153.66) | <0.001 |
| ****Inflammatory variables**** | | | | | |
| ESR (mm/h) | 52.35 (14.00, 75.00) | 52.52 (27.00, 76.25) | 106.38 (22.00, 94.00) | 55.43 (24.50, 91.00) | 0.998 |
| CRP (mg/L) | 19.76 (2.18, 14.40) | 26.03 (2.57, 23.00) | 35.16 (1.94, 57.05) | 27.63 (5.26, 47.83) | 0.578 |
| SII | 806.96 (388.04, 1084.93) | 816.53 (173.34, 1027.39) | 789.50 (314.06, 1181.76) | 637.92 (220.79, 609.89) | 0.635 |
| SIRI | 1.59 (0.27, 2.33) | 1.71 (0.25, 2.18) | 0.86 (0.17, 1.09) | 1.96 (0.87, 2.41) | 0.427 |
| NLR | 5.03 (2.37, 6.87) | 4.19 (1.89, 4.39) | 4.35 (1.99, 6.72) | 3.42 (1.91, 3.22) | 0.548 |
| ****Serology and autoantibodies**** | | | | | |
| IgG (g/L) | 21.53 (13.40, 29.80) | 24.96 (16.73, 29.12) | 19.70 (11.40, 21.80) | 20.54 (14.20, 25.85) | 0.403 |
| IgA (g/L) | 3.57 (2.93, 4.34) | 3.71 (2.58, 4.46) | 2.75 (1.84, 3.74) | 3.54 (2.83, 4.24) | 0.321 |
| IgM (g/L) | 1.23 (0.42, 1.45) | 1.50 (0.97, 2.04) | 1.37 (0.72, 1.38) | 1.61 (0.93, 2.06) | 0.310 |
| C3 (g/L) | 0.66 (0.48, 0.79) | 0.56 (0.36, 0.72) | 0.69 (0.50, 0.88) | 0.64 (0.33, 0.86) | 0.344 |
| C4 (g/L) | 0.14 (0.09, 0.19) | 0.12 (0.05, 0.16) | 0.22 (0.09, 0.28) | 0.14 (0.06, 0.22) | 0.261 |
| ANA titer ≥3200 | 13 (76.5%) | 30 (68.2%) | 7 (53.8%) | 5 (71.4%) | 0.422 |
| Anti-dsDNA (IU/mL) | 107.25 (7.89, 149.00) | 227.94 (8.38, 314.25) | 231.59 (3.30, 328.19) | 116.93 (5.00, 162.00) | 0.948 |
| ANA C1q | 6.78 (0.95, 7.48) | 57.67 (0.75, 14.53) | 11.55 (0.56, 6.05) | 4.77 (1.33, 5.04) | 0.982 |
| Anti-Sm | 13.30 (0.13, 0.34) | 5.27 (0.09, 0.92) | 4.53 (0.09, 0.73) | 35.53 (0.16, 49.55) | 0.745 |
| Anti-nRNP | 8.70 (0.24, 5.76) | 8.44 (0.44, 5.18) | 14.78 (0.11, 4.52) | 28.70 (1.74, 40.99) | 0.848 |
| Anti-SSA | 15.93 (3.15, 3.95) | 11.34 (1.02, 4.24) | 15.90 (0.16, 3.56) | 13.85 (1.14, 3.99) | 0.294 |
| Anti-Ro52 | 26.54 (2.49, 4.76) | 8.90 (0.76, 3.71) | 20.31 (0.10, 4.75) | 17.30 (1.38, 3.00) | 0.271 |
| Anti-SSB | 6.69 (0.07, 1.76) | 1.13 (0.05, 0.73) | 3.95 (0.03, 0.57) | 13.11 (0.06, 0.22) | 0.569 |
| Anti-Scl70 | 0.52 (0.06, 0.42) | 0.50 (0.05, 0.36) | 1.35 (0.05, 0.41) | 0.49 (0.04, 0.58) | 0.992 |
| Anti-PM Scl | 0.20 (0.04, 0.30) | 0.49 (0.07, 0.32) | 0.42 (0.03, 0.53) | 0.28 (0.08, 0.44) | 0.778 |
| Anti-Jo1 | 1.10 (0.08, 0.96) | 0.32 (0.05, 0.37) | 1.27 (0.03, 0.38) | 0.33 (0.11, 0.36) | 0.403 |
| Anti-CENPB | 9.64 (0.01, 0.43) | 0.82 (0.01, 0.31) | 0.44 (0.01, 0.09) | 0.10 (0.03, 0.15) | 0.960 |
| Anti-ANUA | 1.21 (0.22, 0.76) | 4.52 (0.14, 1.74) | 7.26 (0.14, 9.66) | 17.19 (0.36, 1.77) | 0.795 |
| Anti-histone | 0.98 (0.19, 1.02) | 1.25 (0.09, 1.11) | 1.82 (0.10, 1.47) | 3.69 (0.45, 4.25) | 0.270 |
| Anti-RIB P | 1.44 (0.09, 1.41) | 1.06 (0.03, 0.53) | 4.73 (0.04, 0.98) | 11.43 (0.06, 2.61) | 0.608 |
| Anti-AMA M2 | 0.81 (0.06, 0.30) | 1.64 (0.07, 0.74) | 0.51 (0.05, 0.48) | 0.44 (0.28, 0.52) | 0.680 |
| Lupus anticoagulant ratio | 1.22 (1.06, 1.25) | 1.18 (1.02, 1.27) | 1.25 (1.14, 1.33) | 1.09 (0.96, 1.21) | 0.239 |
| aCL IgA | 2.38 (2.50, 2.50) | 4.60 (2.50, 2.50) | 3.63 (2.50, 2.91) | 2.21 (2.50, 2.50) | 0.658 |
| aCL IgG | 3.85 (2.15, 4.70) | 11.92 (3.08, 7.47) | 14.11 (1.90, 5.82) | 9.47 (4.05, 5.35) | 0.195 |
| aCL IgM | 2.56 (2.00, 2.00) | 4.82 (2.00, 2.02) | 4.46 (2.00, 2.00) | 2.54 (2.00, 3.00) | 0.639 |
| Anti β2 glycoprotein IgA | 2.71 (2.00, 2.40) | 3.43 (2.00, 3.55) | 26.64 (2.00, 5.40) | 2.35 (2.00, 2.24) | 0.401 |
| Anti β2 glycoprotein IgG | 3.67 (2.20, 4.80) | 13.05 (2.44, 6.15) | 13.69 (2.00, 3.30) | 3.33 (2.60, 3.60) | 0.471 |
| Anti β2 glycoprotein IgM | 3.62 (2.00, 2.00) | 7.12 (2.00, 2.15) | 7.93 (2.00, 2.08) | 3.85 (2.20, 4.15) | 0.180 |
| **Lymphocyte subsets** | | | | | |
| T cell percent | 73.90 (66.34, 77.40) | 74.55 (65.74, 80.30) | 76.14 (67.16, 84.82) | 74.55 (71.82, 77.50) | 0.784 |
| T helper percent | 29.70 (23.93, 39.57) | 30.50 (21.82, 35.81) | 30.50 (23.72, 31.80) | 35.82 (30.50, 39.06) | 0.541 |
| T suppressor percent | 36.25 (28.10, 45.70) | 37.76 (29.74, 47.18) | 42.10 (37.76, 46.10) | 29.18 (26.50, 37.27) | 0.322 |
| NK cell percent | 11.24 (7.00, 16.00) | 8.94 (5.87, 15.12) | 8.94 (6.91, 15.26) | 3.73 (2.75, 8.52) | 0.307 |
| B cell percent | 10.87 (4.04, 18.15) | 10.87 (5.36, 16.56) | 10.87 (3.50, 11.95) | 15.00 (9.43, 18.31) | 0.558 |
| DPT cell percent | 0.40 (0.16, 0.70) | 0.36 (0.15, 0.54) | 0.36 (0.20, 0.49) | 0.70 (0.34, 0.87) | 0.356 |
| DNT cell percent | 3.70 (2.70, 6.28) | 3.95 (2.94, 5.00) | 3.95 (3.60, 5.93) | 3.95 (3.62, 7.91) | 0.908 |
| NK T cell percent | 0.76 (0.46, 1.71) | 0.76 (0.72, 0.76) | 0.76 (0.76, 1.66) | 0.76 (0.47, 0.76) | 0.408 |
| T cell count | 567.00 (403.00, 992.00) | 719.00 (534.54, 922.50) | 806.00 (719.00, 981.87) | 719.00 (425.21, 1342.50) | 0.442 |
| T helper count | 193.00 (184.90, 381.00) | 260.18 (204.50, 339.25) | 263.00 (260.18, 351.80) | 260.18 (197.13, 632.50) | 0.613 |
| T suppressor count | 326.00 (240.00, 448.00) | 366.98 (214.82, 538.00) | 455.00 (366.98, 621.78) | 359.00 (192.11, 467.99) | 0.547 |
| NK cell count | 88.00 (73.43, 144.00) | 88.00 (50.19, 158.50) | 95.77 (88.00, 107.00) | 43.00 (38.50, 69.89) | 0.159 |
| B cell count | 75.46 (39.00, 106.68) | 75.46 (45.75, 114.50) | 56.00 (38.00, 75.46) | 118.00 (94.90, 340.00) | 0.160 |
| DPT cell count | 2.00 (1.00, 4.00) | 2.00 (2.00, 4.66) | 2.00 (1.49, 4.08) | 5.00 (3.35, 6.50) | 0.158 |
| DNT cell count | 26.00 (18.00, 39.00) | 28.00 (22.50, 50.00) | 28.00 (25.00, 50.00) | 28.00 (17.28, 79.79) | 0.887 |
| NKT cell count | 20.96 (14.00, 21.00) | 20.96 (20.58, 20.96) | 24.88 (20.96, 50.95) | 20.96 (13.13, 20.96) | 0.056 |
| TBNK percent | 98.79 (98.65, 99.12) | 98.79 (98.75, 98.79) | 98.79 (98.07, 98.79) | 98.79 (98.79, 99.61) | 0.524 |
| TBNK count | 767.00 (649.90, 819.90) | 819.90 (819.90, 861.23) | 819.90 (819.90, 1109.31) | 819.90 (574.96, 819.90) | 0.210 |
| THTS ratio | 0.82 (0.65, 1.32) | 0.82 (0.60, 1.20) | 0.71 (0.60, 0.82) | 1.23 (0.82, 1.56) | 0.390 |
| ****Cytokines (pg/mL)**** | | | | | |
| IL-5 | 4.27 (1.32, 2.25) | 2.67 (1.50, 2.79) | 1.86 (1.55, 2.10) | 3.36 (2.12, 2.80) | 0.142 |
| IFN-α | 3.29 (1.74, 2.52) | 4.62 (1.65, 3.42) | 2.04 (1.58, 2.32) | 2.34 (1.46, 3.05) | 0.811 |
| IL-2 | 1.85 (1.54, 1.95) | 2.27 (1.53, 2.62) | 1.60 (1.54, 1.78) | 2.17 (1.80, 2.66) | 0.163 |
| IL-6 | 5.92 (2.10, 8.10) | 7.49 (2.31, 8.34) | 4.65 (2.38, 4.47) | 97.06 (40.81, 104.19) | <0.001 |
| IL-1β | 5.73 (2.22, 6.82) | 6.48 (2.75, 8.44) | 5.66 (3.29, 7.68) | 36.08 (27.52, 42.45) | <0.001 |
| IL-10 | 2.18 (1.63, 2.60) | 4.64 (1.72, 3.42) | 2.03 (1.76, 2.26) | 2.79 (2.00, 3.67) | 0.407 |
| IFN-γ | 7.33 (2.16, 7.94) | 12.24 (2.84, 14.70) | 24.82 (5.75, 15.32) | 7.61 (2.11, 5.78) | 0.400 |
| IL-8 | 20.08 (2.10, 8.43) | 10.58 (3.02, 10.38) | 3.78 (2.10, 4.06) | 5.73 (2.84, 8.43) | 0.280 |
| IL-17 | 5.04 (1.65, 3.66) | 5.97 (2.45, 5.82) | 3.93 (2.04, 4.31) | 4.81 (2.96, 5.25) | 0.378 |
| IL-4 | 1.29 (0.93, 1.63) | 1.52 (1.10, 2.01) | 1.24 (0.83, 1.45) | 1.30 (1.20, 1.46) | 0.629 |
| IL-12p70 | 2.53 (1.32, 1.97) | 1.87 (1.41, 2.25) | 1.61 (1.28, 1.66) | 1.93 (1.52, 2.06) | 0.483 |
| TNF-α | 2.24 (2.04, 2.21) | 2.28 (1.65, 2.66) | 2.45 (2.05, 2.21) | 12.99 (10.27, 14.55) | <0.001 |
| IL-5 | 4.27 (1.32, 2.25) | 2.67 (1.50, 2.79) | 1.86 (1.55, 2.10) | 3.36 (2.12, 2.80) | 0.142 |
| IFN-α | 3.29 (1.74, 2.52) | 4.62 (1.65, 3.42) | 2.04 (1.58, 2.32) | 2.34 (1.46, 3.05) | 0.811 |
| ****Pulmonary function tests**** | | | | | |
| FVC percent | 64.82 (57.00, 72.80) | 68.92 (67.38, 71.98) | 71.41 (65.60, 67.50) | 66.76 (67.50, 67.50) | 0.932 |
| FEV1 | 69.19 (63.00, 79.80) | 64.01 (66.16, 69.25) | 71.28 (66.50, 67.55) | 68.05 (67.55, 67.55) | 1.000 |
| TLC | 76.88 (78.15, 79.50) | 72.94 (76.60, 78.15) | 83.40 (78.15, 86.10) | 88.38 (78.15, 94.58) | 0.318 |
| DLCO | 48.93 (49.80, 49.80) | 46.56 (47.12, 49.80) | 51.91 (49.80, 53.20) | 54.73 (49.80, 56.10) | 0.429 |
| ****Treatment**** | | | | | |
| Glucocorticoids | 18 (100.0) | 38 (95.0) | 15 (100.0) | 7 (87.5) | 0.232 |
| Dose classification |  |  |  |  | 0.045 |
| High dose | 8 (44.4) | 17 (42.5) | 6 (40.0) | 4 (50.0) |  |
| Medium dose | 5 (27.8) | 16 (40.0) | 5 (33.3) | 3 (37.5) |  |
| Low dose | 5 (27.8) | 5 (12.5) | 4 (26.7) | 0 (0.0) |  |
| ****Immunosuppressants**** | | | | | |
| Hydroxychloroquine sulfate | 15 (83.3) | 35 (87.5) | 12 (80.0) | 6 (75.0) | 0.772 |
| Cyclophosphamide | 9 (50.0) | 8 (20.0) | 4 (26.7) | 4 (50.0) | 0.077 |
| Mycophenolate mofetil | 9 (50.0) | 14 (35.0) | 6 (40.0) | 1 (12.5) | 0.286 |
| Ciclosporin A | 1 (5.6) | 2 (5.0) | 1 (6.7) | 1 (12.5) | 0.892 |
| Tacrolimus | 1 (5.6) | 2 (5.0) | 2 (13.3) | 0 (0.0) | 0.649 |
| Azathioprine | 0 (0.0) | 2 (5.0) | 1 (6.7) | 1 (12.5) | 0.520 |
| Methotrexate | 1 (5.6) | 1 (2.5) | 0 (0.0) | 0 (0.0) | 0.703 |
| Iguratimod | 1 (5.6) | 0 (0.0) | 0 (0.0) | 0 (0.0) | 0.180 |
| ****PAH treatment regimen**** | | | | | |
| Endothelin  receptor antagonist | 7 (38.9) | 10 (25.0) | 5 (33.3) | 1 (12.5) | 0.510 |
| Phosphodiesterase 5 inhibitor | 5 (27.8) | 9 (22.5) | 6 (40.0) | 2 (25.0) | 0.649 |
| Prostaglandin analogs | 0 (0.0) | 2 (5.0) | 0 (0.0) | 0 (0.0) | 0.676 |

Abbreviations: BMI, body mass index; mPAP, mean pulmonary artery pressure; 6MWD, 6-minute walking distance; SLEDAI, Systemic Lupus Erythematosus Disease Activity Index; TTP, thrombotic thrombocytopenic purpura; ILD, interstitial lung disease; WBC, white blood cell count; Hb, hemoglobin; PLT, platelet; AST, aspartate aminotransferase; ALT, alanine aminotransferase; LDH, lactate dehydrogenase; eGFR, estimated glomerular filtration rate; BNP, brain natriuretic peptide; ESR, erythrocyte sedimentation rate; CRP, C-reactive protein; SII, systemic immune-inflammation index; SIRI, systemic inflammation response index; NLR, neutrophil-to-lymphocyte ratio; Ig, immunoglobulin; C3, complement 3; C4, complement 4; ANA, antinuclear antibody; anti-dsDNA, anti-double-stranded DNA antibody; anti-Sm, anti-Smith antibody; anti-nRNP, anti-ribonucleoprotein antibody; anti-SSA, anti-Sjögren's syndrome antigen A antibody; anti-Ro52, anti-Ro52 antibody; anti-SSB, anti-Sjögren's syndrome antigen B antibody; anti-Scl70, anti-topoisomerase I antibody; anti-PM-Scl, anti-polymyositis-scleroderma antibody; anti-Jo1, anti-histidyl-tRNA synthetase antibody; anti-CENPB, anti-centromere protein B antibody; anti-ANUA, anti-nucleosome antibody; anti-RIB P, anti-ribosomal P protein antibody; anti-AMA-M2, anti-mitochondrial antibody M2; aCL, anticardiolipin antibody; anti-β2GPI, anti-β2 glycoprotein I antibody; NK cell, natural killer cell; DPT cell, double-positive T cell; DNT cell, double-negative T cell; NKT cell, natural killer T cell; TBNK, T cells, B cells, and NK cells; THTS, T helper/T suppressor ratio; IL, interleukin; IFN, interferon; TNF, tumor necrosis factor; FVC, forced vital capacity; FEV1, forced expiratory volume in 1 second; TLC, total lung capacity; DLCO, diffusing capacity of the lung for carbon monoxide; MMF, mycophenolate mofetil; ERA, endothelin receptor antagonist; PDE5i, phosphodiesterase type 5 inhibitor.

**Supplementary Table 5. Univariate Cox Regression Analysis for Mortality in SLE-PAH Patients**

| **Predictor variable** | **HR (95% CI)** | **P value** |
| --- | --- | --- |
| Trajectory 4 (vs 1) | 13.697 (3.392–55.305) | 0.025 |
| SIRI | 1.399 (1.069–1.831) | 0.014 |
| TNF-α | 1.338 (1.174–1.525) | <0.001 |
| SLEDAI | 1.214 (1.092–1.349) | <0.001 |
| IL-1β | 1.070 (1.031–1.109) | <0.001 |
| Age | 1.060 (1.012–1.110) | 0.013 |
| Anti-CENPB | 1.029 (1.010–1.047) | 0.002 |
| IL-6 | 1.025 (1.011–1.039) | <0.001 |
| BNP | 1.001 (1.001–1.002) | <0.001 |
| 6MWD | 0.818 (0.715–0.937) | 0.004 |
| Gender (female vs male) | 0.145 (0.029–0.741) | 0.020 |

Abbreviations: HR , hazard ratio; 95% CI ,95% confidence interval;SIRI, systemic inflammation response index; TNF-α, tumor necrosis factor-alpha; SLEDAI, Systemic Lupus Erythematosus Disease Activity Index; IL-1β, interleukin-1 beta; Age, age at baseline; Anti-CENPB, anti-centromere protein B antibody; IL-6, interleukin-6; BNP, brain natriuretic peptide; 6MWD, 6-minute walking distance.

**Supplementary Table 6. Multivariable Cox Regression Analysis for Mortality in SLE-PAH Patients.**

| **Predictor variable** | **β coefficient** | **HR (95% CI)** | **P value** |
| --- | --- | --- | --- |
| Trajectory 4 (vs 1) | 6.188 | 8.843 (1.441–16.314) | **0.037** |
| SLEDAI | 0.407 | 1.502 (1.036–2.177) | **0.032** |
| 6MWD | -0.230 | 0.795 (0.647–0.976) | **0.028** |
| IL-6 | 0.030 | 1.031 (1.003–1.059) | **0.029** |
| SIRI | 0.603 | 1.828 (1.104–3.026) | **0.019** |
| Gender (female vs male) | 2.398 | 11.001 (0.021–5888.173) | 0.454 |
| Anti-CENPB | 0.020 | 1.020 (0.973–1.070) | 0.407 |

Abbreviations: HR , hazard ratio; 95% CI ,95% confidence interval; SLEDAI, Systemic Lupus Erythematosus Disease Activity Index; 6MWD, 6-minute walking distance; IL-6, interleukin-6; SIRI, systemic inflammation response index; Anti-CENPB, anti-centromere protein B antibody.
